# A new marker constructed from immune-related lncRNA pairs can be used to predict clinical treatment effects and prognosis: in-depth exploration of underlying mechanisms in HNSCC

**DOI:** 10.1186/s12957-023-03066-x

**Published:** 2023-08-17

**Authors:** Xin Fan, Yuhan Huang, Yun Zhong, Yujie Yan, Jiaqi Li, Yanting Fan, Fei Xie, Qing Luo, Zhiyuan Zhang

**Affiliations:** 1 Department of Otolaryngology Head and Neck Surgery, The First Affiliated Hospital of Nanchang University, Nanchang, Jiangxi Province China; 2 Yunnan University of Chinese Medicine, Kunming, Yunnan Province China; 3 The First Clinical Medical College of Nanchang University, Nanchang, Jiangxi Province China; 4 School of Stomatology, Nanchang University, Nanchang, Jiangxi Province China

**Keywords:** Clinical application value, Head and neck squamous cell carcinoma, Immune-related lncRNA pairs, Marker, Potential mechanism

## Abstract

**Background:**

Long non-coding RNA (lncRNA) plays a vital role in tumor proliferation, migration, and treatment. Since it is challenging to standardize the gene expression levels detected by different platforms, the signatures composed of many immune-related single lncRNAs are still inaccurate. Utilizing a gene pair formed of two immune-related lncRNAs and strategically assigning values can effectively meet the demand for a higher-accuracy dual biomarker combination.

**Methods:**

Co-expression and differential expression analyses were performed on immune genes and lncRNAs data from The Cancer Genome Atlas and the ImmPort database to obtain differentially expressed immune-related lncRNAs for pairwise pairing. The prognostic-related differentially expressed immune-related lncRNAs (PR-DE-irlncRNAs) pairs were then identified by univariate Cox regression and used for lasso regression to construct a prognostic model. Various methods were used to validate the predictive prognostic performance of the model. Additionally, we explored the potential guiding value of the model in immunotherapy and chemotherapy and constructed a nomogram suitable for efficient prognosis prediction. Mechanistic exploration of anti-tumor immunity and mutational perspectives are also included. We also analyzed the correlation between the model and immune checkpoint inhibitors (ICIs)-related, N6-methyadenosine (m6A)-related, and multidrug resistance genes.

**Results:**

We used a total of 20 pairs of PR-DE-irlncRNAs to create a prognosis model. Quantitative real-time polymerase chain reaction experiments further verified the abnormal expression of 11 lncRNAs in HNSCC cells. Various methods have confirmed the excellent performance of the model in predicting patient prognosis. We reasoned that lncRNAs/TP53 mutation might play a positive/negative anti-tumor role through the immune system by multi-perspective analyses. Finally, it was found that the prognostic model was closely related to immunotherapy and chemotherapy as well as the expression of ICIs/m6A/multidrug resistance-related genes.

**Conclusion:**

The prognostic model performs excellently in predicting the prognosis of patients and provides the potential value of practical guidance for treatment.

**Supplementary Information:**

The online version contains supplementary material available at 10.1186/s12957-023-03066-x.

## Introduction

In advanced stages of head and neck squamous cell carcinoma (HNSCC), an aggressive malignancy, mortality, and morbidity are high [1]. With approximately 800,000 new cases and 430,000 new deaths worldwide each year, HNSCC is one of the most common causes of cancer-related deaths [2, 3]. Surgery, radiotherapy, chemotherapy, and combination therapy are still the standard treatment methods for HNSCC [4]. Unfortunately, due to the frequent local and distant metastasis of HNSCC and the resistance to chemotherapeutics, there is currently no entirely satisfactory treatment for advanced HNSCC, which leads to a high mortality rate in patients [5, 6]. Although in recent years, the clinical application of immune checkpoint inhibitors (ICIs), such as nivolumab and pembrolizumab, has completely changed the treatment outcome of metastatic or recurrent HNSCC. However, its objective response rate is still 20%. The risk of immune-related adverse events (irAEs) that can contribute to severe or fatal toxicities for patients hinder the wide application of ICI therapy [7–9]. In the combination therapy of ICIs, the incidence of side effects is higher than that of ICIs monotherapy, and the side effects also occur faster [10, 11]. In addition, targeting CD44, a marker for cancer stem cell-like cells (CSCs), has recently been regarded as a promising therapeutic target for HNSCC treatment; however, further clinical applications are still being explored [12, 13]. As a type of RNA without protein-coding ability, long-chain non-coding RNA (lncRNA) not only participates in gene regulation processes, such as regulating mRNA splicing, chromatin, histone remodeling, and transcription regulation [14], but also participates in biological regulation processes such as tumor occurrence, development, and metastasis [15, 16]. Increasing evidence shows that lncRNAs may play a vital role in the proliferation, migration, and treatment of HNSCC [17–20]. In recent years, lncRNAs have been identified to facilitate the resistance to cisplatin, paclitaxel, 5FU, and other chemotherapeutic drugs in various ways [21–25]. Overexpression of lncRNA-UCA1 can protect the expression of PDL1 from miRNAs, thereby upregulating the expression of PDL1 and ultimately promoting the immune escape of GC cells [26]. Given higher tissue specificity and easier detection than mRNA, lncRNA is more suitable as a biomarker for tumor diagnosis and prognosis [27, 28]. Consequently, a growing number of studies note that ir-lncRNAs signals can predict the prognosis and treatment sensitivity of various cancers, such as melanoma, lung adenocarcinoma, and endometrial cancer [29–31]. Unfortunately, most of these predictive signatures are combinations of single lncRNAs. In contrast, the dual biomarker combination is superior to a single marker in terms of the accuracy of the cancer diagnosis model [32]. To achieve higher accuracy, it is indispensable to develop a model based on the combination of double lncRNAs for HNSCC prognosis. Due to the technical differences between different platforms, it is difficult for the detected gene expression levels to be of the same standard [33]. Recently, novel gene pair signatures have been developed to circumvent this problem subtly. By comparing the expression of two genes in each patient, the researchers assigned a value of 1 (expression of gene A >expression of gene B) or 0 (expression of gene A <expression of gene B) to this gene pair [33]. It is evident that such a combination of lncRNAs meets the need for a dual biomarker combination with higher accuracy.

LncRNAs have been demonstrated to regulate cancer progression through immune regulation, mainly by changing the immune microenvironment. Xiong et al. found that with the upregulation of lncRNA-POU3F3 expression in cancer-related cells, circulating regulatory T cells (Tregs) increased in gastric cancer patients [34]. In vitro experiments further confirmed that lncRNA-POU3F3 promoted Treg differentiation by activating TGFβ signaling, thereby promoting the proliferation of tumor cells [34]. In hepatocellular carcinoma (HCC), Jiang et al. also found that the highly expressed lncRNA-EGFR stimulated Treg production and continuous activation, resulting in the suppression of cytotoxic T cells [35]. In addition, RP11-284N8.3.1 and AC104699.1.1 are related to T cell activation and differentiation and are associated with the increasing survival rate of ovarian cancer [36].

We aspire to obtain differentially expressed immune-related lncRNAs (DE-irlncRNAs) by combining the HNSCC RNA sequencing data obtained from the TCGA database and the immune-related genes obtained from the ImmPort database. The effective paired DE-irlncRNAs were then screened to find the prognostic-related DE-irlncRNAs pairs (PR-DE-irlncRNAs pairs). Following this, a prognostic model was designed based on 20 pairs of PR-DE-irlncRNAs, and the sample risk score was calculated. We performed Risk plots, Receiver Operating Characteristic (ROC) curves, Kaplan–Meier (K-M) curves, Cox regression analysis, and clinical features subgroup analysis to verify the accuracy of the model’s predictive ability. In parallel, we investigated their possible mechanisms of action in HNSCC by gene set enrichment analysis (GSEA), tumor immune microenvironment (TIME) analysis, and mutation analysis. Finally, to determine whether prognostic models can be applied to treatment guidance and prognosis evaluation for patients with HNSCC, immunophenotypic score (IPS) analysis, drug sensitivity analysis, ICIs/m6A/multidrug resistance-related genes analysis and nomogram were used.

## Methods

### Data collection and collation

The transcription component data of 502 HNSCC samples and 44 adjacent normal tissues were downloaded from The Cancer Genome Atlas (TCGA) database. Tumor and normal oral tissue were not paired. Simultaneously, the clinical data (including overall survival, vital status, age, gender, grade, stage, T stage, and N stage) were obtained in an identical manner. For the following analysis, 2483 immune-related genes (IRGs) were extracted from the ImmPort database.

### Differential expression analysis and pairing of ir-lncRNAs obtained by co-expression analysis

By co-expression analysis, the threshold was set to the correlation coefficient >0.5 and *p* value < 0.001. The expression values of 1718 IRGs and 14,086 lncRNAs extracted from the expression matrix of TCGA were used to filter irlncRNAs. The R package “limma” was then applied to the differential expression of irlncRNAs between 504 HNSCC samples and 44 normal tissues to identify the DE-irlncRNAs according to the filtering condition that was set to |log2FC (fold-change)| >1 and FDR <0.001 [37]. The lncRNA pairs consist of two DE-irlncRNAs that were paired recurrently singly with each other. We defined lncRNA pair K as a comparison of the expression levels of lncRNA *i* and lncRNA *j*. The *K* equals 1 if the expression level of lncRNA *i* is higher than that of lncRNA *j*, and the reverse is *K* = 0. These values of lncRNA pairs *K* were used for further filtering for effectively matched lncRNA pairs. The lncRNA pairs *K* defined as 0 or 1 could be identified as effectively matched lncRNA pairs for the subsequent analysis on the condition that their numbers accounted for more than 20% and less than 80% of all samples.

### Construction and evaluation of prognostic model

After integration, to filter for PR-DE-irlncRNAs pairs, we analyzed the data of HNSCC samples with complete overall survival (OS) and effectively matched lncRNA pairs by univariate Cox analysis (*p* value <0.001); 499 samples with complete OS data and defined values of PR-DE-irlncRNAs pairs were randomly assigned to the training set (*n* = 300) and the validation set (*n* = 199) at a ratio of 6:4 for the subsequent analysis. To screen out highly correlated PR-DE-irlncRNAs pairs, the lasso regression was used to analyze defined values of 97 PR-DE-irlncRNAs pairs, which could minimize the risk of overfitting for screening signatures. Finally, the optimal penalty parameter (*λ*) determined by the minimum 10-fold cross-validation was employed to construct a prognostic model based on 20 PR-DE-irlncRNAs pairs. After optimizing the model, the formula for calculating the risk score of the sample was listed as follows:

$$\mathrm{Risk\ score}={\sum }_{\mathrm{i}=1}^{\mathrm{n}}\mathrm{corresponding\ coefficient }\times \mathrm{PR}-\mathrm{DE}-\mathrm{irlncRNAs\ pair}{\mathrm{s}}^{\mathrm{^{\prime}}}\mathrm{defined\ value}$$

Following the risk score of each sample calculation, the HNSCC samples from the three sets were respectively divided into the high-risk group and the low-risk group according to the median risk score of each set. Firstly, we used the risk curve and survival status graph to show the contact between the patient's risk score and survival status. The R package “SurvivalROC,” with the capability to plot muti-ROC curves containing other clinical factors, was used to draw ROC curves of risk score, which could evaluate the accuracy and optimality of the model in predicting the sample’s survival. In addition, we performed K-M analysis by R packages “survival” and “survminer” to compare the sample’s survival differences between the high-risk and low-risk groups [38], and these results were visualized by the survival curve. To detect whether the risk score could be used as an independent prognostic indicator of survival, we used univariate and multivariate Cox regression to analyze the relationship between available variables (age, gender, grade, stage, T, N, and risk score) and OS.

### Assess the relationship between risk score and clinical characteristics

With the purpose of assessing the relationship between the clinical characteristics and the risk score derived from the prognostic model, we conducted the chi-square test and used the heatmap to visualize the distribution of the clinical characteristics of every sample between the high-risk and low-risk groups within the whole set. Moreover, to compare differences in the risk score among different groups of these clinical features, we utilized the Mann–Whitney *U* test for visualization. R package “ComplexHeatmap,” “limma,” and “ggpubr” were used in these two analyses [39]. Additionally, we used the K-M test to identify whether the prognostic model retained the ability to predict the OS in each subgroup with different clinical characteristics.

### Gene set enrichment analysis

We applied GO and KEGG enrichment analyses for the differential expressed genes (DEGs) between the high-risk and the low-risk groups to investigate the biological functions and pathways related to the prognostic model. We set the threshold to |log2FC| ≥1 and FDR <0.05 to filter out DEGs. In addition to utilizing the Gene Ontology (GO) for investigating possible biological functions, the Kyoto Encyclopedia of Genes and Genomes (KEGG) was also used to identify possible pathways involved in DEGs. The research was conducted by the R package “clusterProfiler,” “org.Hs.eg.db,” and “enrichplot,” with corresponding results displayed in bar plots and bubble graphs [40].

### Evaluate the relationship between risk score and immune response

To evaluate the correlation between immune cells/stromal cells and risk score, the R package “estimate” was used to calculate immune and stromal cell scores for each sample. We then compared the difference in immune and stromal cell scores of patients between the high-risk and low-risk groups. Moreover, Spearman correlation analysis was used to analyze the correlation between immune/stromal cells score and risk score. To further explore the relationship between tumor-infiltrating immune cells and the prognostic model, we used the immune infiltrating cell content of each HNSCC sample calculated by the five most advanced algorithms (including TIMER, CIBERSORT, XCELL, QUANTISEQ, and EPIC) from the TIMER 2.0 database (timer.comp-genomics.org). We applied Spearman correlation analysis to evaluate the connection between risk score and immune infiltrating cells and used the point graph to visualize the correlation results. Only the results with the significant correlation (*p* <0.05) were demonstrated. Mann–Whitney *U* test analysis was utilized to compare the content of immune infiltrating cells between high-risk and low-risk groups, with final results displayed in violin plots. The R packages “ggplot2,” “scales,” “ggtext,” and “vioplot” were used in the aforementioned analyses [40]. Besides, the single-sample gene set enrichment analysis (ssGSEA) was conducted using the R packages “GSEABase” and “gsva” to further quantify the scores of 16 immune cells and 13 immune functions, enabling exploration of their relationship with risk score. Spearman correlation and Mann–Whitney *U* test analyses were also used during this process.

### Mutational analysis

The somatic gene mutation data of HNSCC samples were downloaded from the TCGA database to explore the relationship between gene mutations and the prognostic model. VarScan was used to examine MAF files of somatic mutations, while the R package “GenVisR” was employed to visualize the 30 most frequently mutated genes in both high-risk and low-risk groups [41]. Tumor mutation burden (TMB), defined as the number of somatic cells, coding, indel mutations, and base substitutions in a million bases in the genome [42], was calculated by Perl software. To explore the effect of TMB on survival, we used K-M analysis to compare the OS difference between the high TMB and low TMB groups. After stratifying TCGA samples based on the TP53 mutation state into wild and mutant groups, we compared the difference in the risk score between the TP53 mutant and the TP53 wild groups, thus exploring the association of TP53 mutation with immune response. Additionally, we used the K-M analysis to compare the differences in OS between the two groups. To explore the association between TP53 mutation state and immune infiltrating cells, we applied the Cibersort deconvolution algorithm to obtain matrix data of the ratio of 22 immune cells in each tumor sample based on RNA sequencing data according to the filter threshold of *p* < 0.05 [43]. We compared and visualized immune cell content between the two groups using R packages “Limma” and “Vioplot” [44] and also observed the expression difference of PDL1 (CD274) between the two groups due to the significant role of TP53 mutation in predicting the effectiveness of PD1/PDL1.

### Explore the application of the prognostic model in immunotherapy

Given the critical role of ICIs in immunotherapy, the Spearman correlation analysis was run to explore the connection between risk score and expression of ICIs-related genes. In addition, we compared the difference in ICIs-related genes’ expression of samples between high-risk and low-risk groups to validate the correlation results. Analogous methods were also applied to m6A-related genes and multidrug resistance genes, such as MRP1 (ABCC1) and MRP3 (ABCC3), which probed their correlation with the prognostic model. R packages “ggplot2” and “reshape2” were included in the analysis [45].

We downloaded the IPS from the Atlas of Cancer Immunity (TCIA) database. IPS of the patients was obtained by evaluating the gene expression of four cell types (including effector cells, immunosuppressive cells, MHC molecules, and immunomodulators) that determined immunogenicity [46]. Spearman correlation analysis was operated to evaluate the correlation between risk score and four types of IPS, as well as to compare the difference in IPS between the high-risk and low-risk groups. The R package “pRRophetic” was used to predict the half-inhibitory concentration (IC50) of chemotherapeutics recommended by the NCCN guidelines for treating each sample of HNSCC [47]. In addition to the correlation between the IC50 of each drug and the risk score, the IC50 difference between high-risk and low-risk populations was also analyzed. To predict the IC50 of the drug in this R package, we applied the cell line expression data from the Cancer Drug Sensitivity Genomics (GDSC) database and RNA sequencing transcriptome data from the TCGA database to construct the ridge regression model [48].

### Construction and verification of Nomogram

A multivariate Cox regression model consisted of risk scores and clinical factors that were significantly related to prognosis, as filtered out by univariate Cox regression, in order to create a more clinically appropriate quantitative tool for predicting the 1-, 2-, and 3-year OS. The final result was presented as Nomogram, while the calibration curve was used to estimate the accuracy of survival prediction. In addition, we employed the multi-factor ROC curve to verify the accuracy of the Nomogram and optimality in predicting the 1-, 2-, and 3-year OS. In this process, the R packages “rms,” “survival,” and “survivalROC” were used.

### Validation of abnormal expression of modeled genes in HNSCC cells

We purchased human oral squamous cell carcinoma cells (scc9 and cal27) from Shanghai Anwei Biotechnology Co., Ltd. In addition, we purchased normal oral gingival epithelial cell line (HEG) from Shanghai Baiye Biotechnology Center. We cultured these three cells in complete DMEM (Gibco, Cat#C11995500BT) or DMEM/F12 (Gibco, Cat#C11330500BT) containing 10% fetal bovine serum (Excell, Cat#FSP500) and 1% penicillin-streptomycin liquid (Solarbio, Cat#P1400). All cells were cultured at 37 °C in 5% CO2’s humidified incubator.

The 20 PR-DE-irlncRNA pairs used for modeling comprised of 35 lncRNAs. We endeavored to design primers for these lncRNAs required for QRT-PCR (quantitative real-time polymerase chain reaction) experiments. Ultimately, we only succeeded in designing primers for 14 lncRNAs that can be used effectively in QRT-PCR. Table 1 listed the primer sequences of 15 lncRNAs. Total RNA was extracted and purified from three cells using TransZol Up Plus RNA Kit (TRANS, Beijing, China), followed by cDNA synthesis with HiScript® III RT SuperMix for qPCR (+gDNA wiper) (Vazyme, Nanjing, China) according to the manufacturer’s instructions. QRT-PCR was performed using the QuantStudio™ 3 96 Real-time Fluorescent Quantitative PCR System (Applied Biosystems, Waltham, Massachusetts, USA) and Taq Pro Universal SYBR qPCR Master Mix (Vazyme, Nanjing, China). After normalizing all measured values to relative expression levels of β-actin via the 2^−ΔΔCt^ method, we compared differences in the expression levels of 15 lncRNAs between cal27/scc9 and HEG cells.

**Table 1:** All primer sequences used in QRT-PCR experiment

Gene	Forward primer	Reverse primer
β-Actin	CTGTAGAGAAGAGGAACCGTAGC	TGGTTGACCTAGAAATGGAAGGAA
AC098487.1	CAGAACCTACGCACCTACG	CCGTCTACACTGGAAGCAG
MIR924HG	ACCACCGAGTTGACAAAAGT	GCTGCTGGAGGTTTACTTGA
LINC00944	CCTCTTAATCCTCTGTCCTCCATC	CTCTCCAGTGTTATGAAGTTCAAGT
RUSC1-AS1	TGCATTTGTTGTCCTGGATG	GCTGGTTTCAGGGTACAGGA
LINC00205	GGCTTTTGTGCCTGGAAGTG	GGGAAGTTCTGAGCTGGCAT
SNHG25	GCAGGTTCCGGGAGGTCA	CAAACCACTTTATTGACGGGAA
PTOV1-AS2	CGG​CAC​TAG​GGA​AAC​GTC​AT	TGT​CCA​CCG​ATG​ATC​TCC​CT
KDM4A-AS1	TTGCCTGGATGGCTGAGAATC	TTCCTTTCACCCTCCTTCCTTC
C5orf66-AS1	CGGGATCAACCCTCTGCTTT	TTCTTGAGAAGCGACTGCGT
TMPO-AS1	AGCGACAAGATCCCTTTCATTC	CGTTGCCGGACTTCACCTT
AL354733.3	GTCATTGGCGTTCGTGGATG	TGTGAAAACCCTGGTTGGCT
HOXC-AS1	CAACTCCATCTCTGCGACAC	AACAAGCTACTTGCCCACGA
AL390719.2	ATGGGATAGGGAAGGCAGGT	CAGGGTCCTTCCTGTCACAC
AL133243.2	AGTCCACCATTGCTCAACCGA	ATCGGCCTTACATCTCCTGGC

### Statistical analysis

According to the distribution characteristics, Student’s *t*-test or Mann–Whitney *U* test was utilized to compare continuous variables. At the same time, the chi-square test or Fisher exact test was used to compare categorical variables. DE-irlncRNAs pairs related to prognosis, which univariate Cox regression analysis confirmed, were used to screen out the optimal genes to construct the prognostic model by lasso regression. Multivariate Cox regression was used to construct the Nomogram. The study employed the K-M curve with the log-rank test to compare survival between different groups, while the ROC curve was used to evaluate the prognostic predictive ability of each factor. The univariate and multivariate Cox regression analyses were applied to evaluate the independent predictive value of risk scores. Spearman or Pearson was used for analyzing the correlation between two variables. We performed all analyses in our study in R programming language (version 4.0.3), Perl (version 5.32.1001), and SPSS Statistics software 22. All statistical *p* values were two-tailed, and the results identified as statistically significant had the threshold of the *p* value <0.05 or FDR <0.05.

## Results

### Data acquisition

**Fig. 1:**
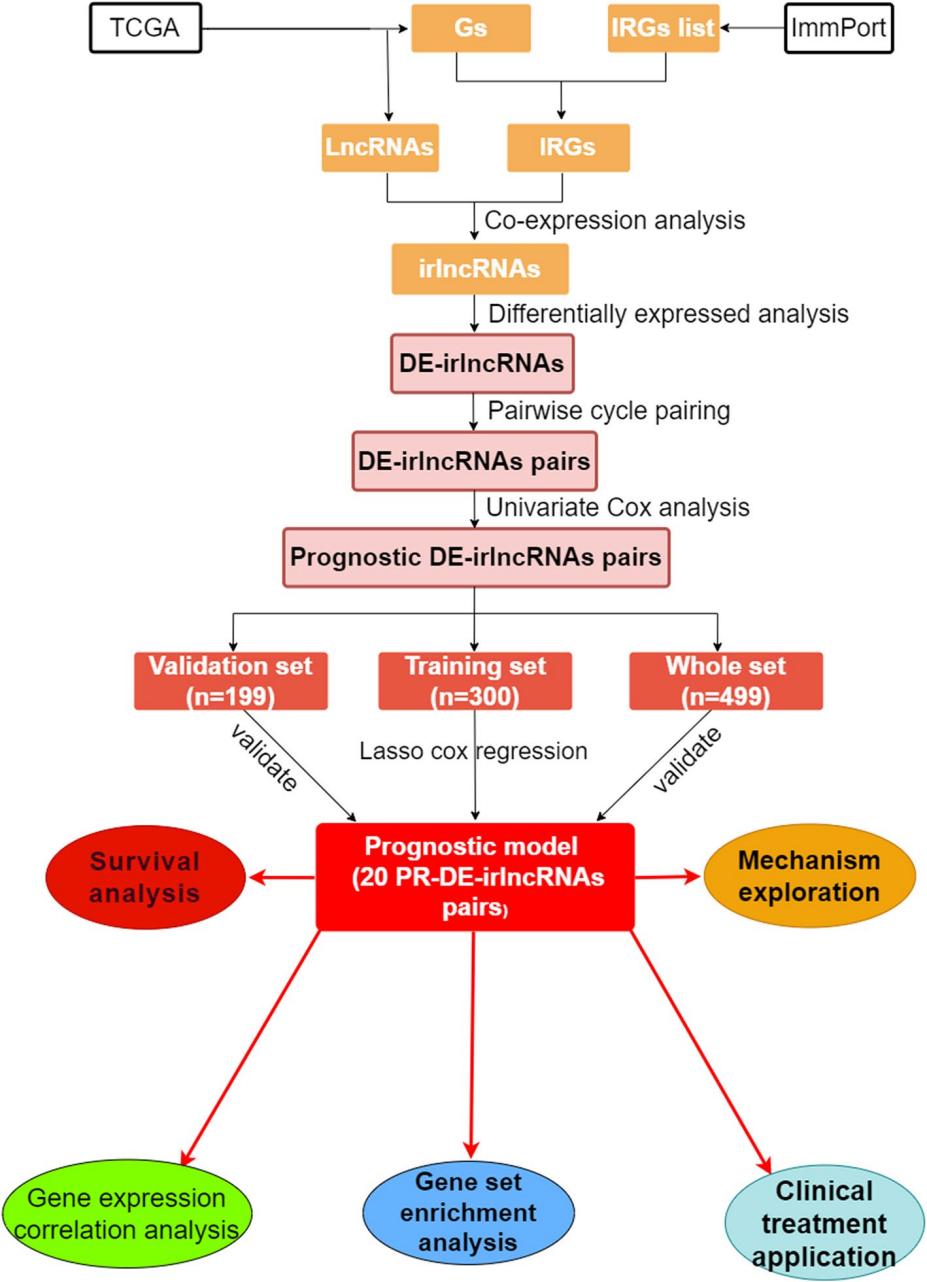
Flow chart of prognostic model construction and verification

**Fig. 2:**
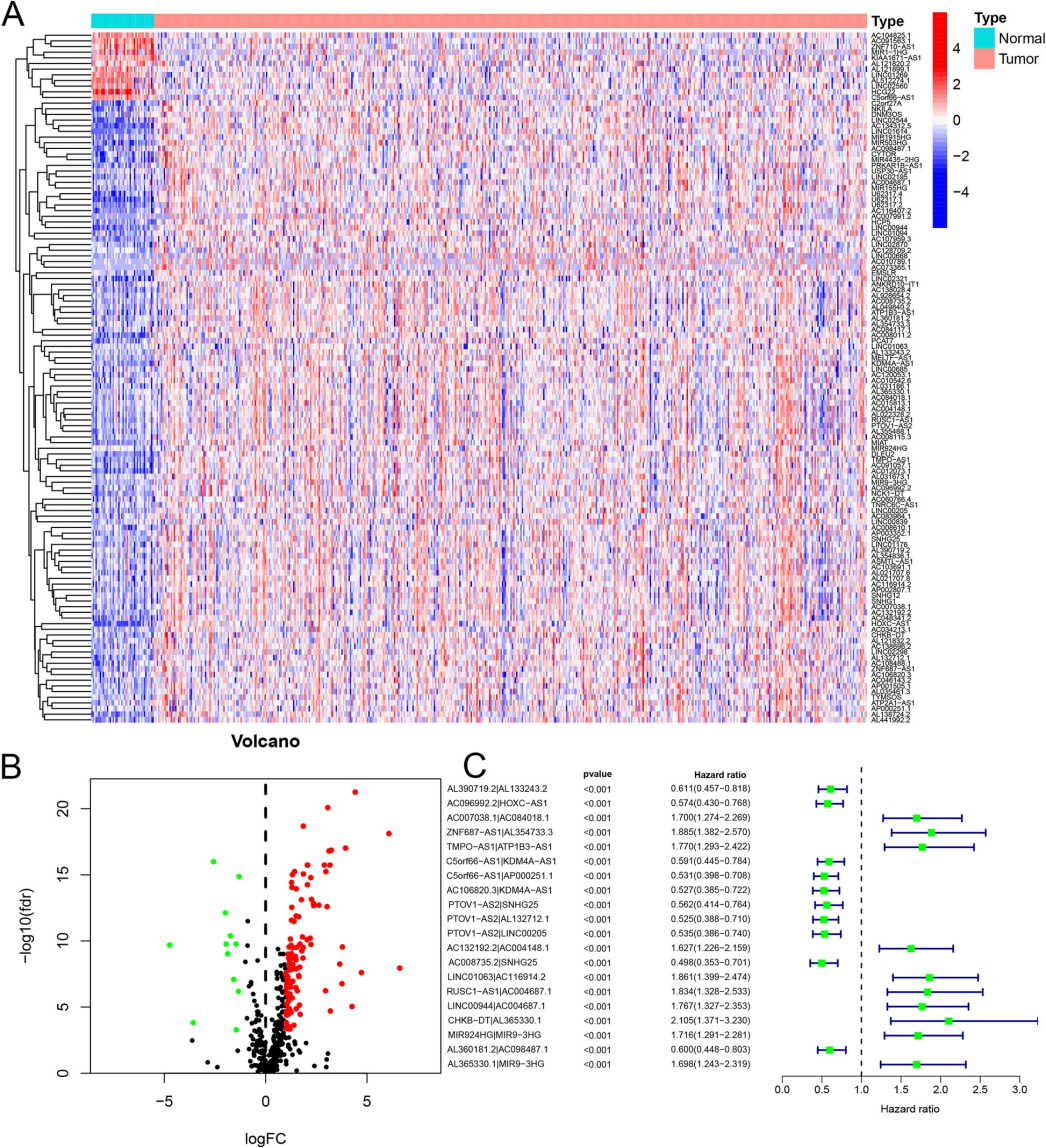
Description of DE-irlncRNAs and PR-DE-irlncRNAs pairs. **A** The heat map demonstrated the expression profile of DE-irlncRNAs. **B** The volcano chart revealed the regulation profile of DE-irlncRNAs. **C** The results of univariate Cox regression analysis on the basis of 20 PR-DE-irlncRNAs pairs and survival rate were displayed in the forest plot

In Fig. 1, we show the flow chart outlining the prognosis model making. We identified 442 ir-lncRNAs under the condition of the co-expression analysis on RNA sequencing data of IRGs and lncRNAs. Subsequent differential expression analysis of the expression levels of 442 ir-lncRNAs revealed significant differences (Fig. 2A), with the expression of 110 ir-lncRNAs increased and 12 decreased (Fig. 2B).

### Construction of prognostic model and analysis of corresponding PR-DE-ir-lncRNAs pairs

Through integrating mRNA expression and clinical data of HNSCC patients, we acquired the clinical features of 499 HNSCC samples shown in Table 2. We filtered out 97 PR-DE-irlncRNAs pairs through operating univariate Cox analysis based on OS. Our study employed lasso regression analysis to construct the prognostic model using the data from the training set. Finally, 20 PR-DE-irlncRNAs pairs were identified based on the optimum value of *λ* (Fig. S1). Table 3 showed 20 PR-DE-irlncRNAs pairs, along with their corresponding coefficients in the model for calculating the risk score of each sample. AC007038.1|AC084018.1, ZNF687−AS1|AL354733.3, TMPO−AS1|ATP1B3−AS1, AC132192.2|AC004148.1, LINC01063|AC116914.2, RUSC1−AS1 |AC004687.1, LINC00944|AC004687.1, CHKB−DT|AL365330.1, MIR924HG|MIR9−3HG, and AL365330.1|MIR9−3HG (HR >1.0) shown in Fig. 2C played negative significant roles, while AL390719.2|AL133243.2, AC096992.2|HOXC-AS1, C5orf66-AS1|KDM4A-AS1, C5orf66-AS1|AP000251.1, AC106820.3|KDM4A-AS1, PTOV1-AS2|SNHG25, PTOV1-AS2|AL132712.1, PTOV1-AS2|LINC00205, AC008735.2|SNHG25, and AL360181.2|AC098487.1 (HR <1.0) played positive roles in survival.

**Table 2:** Clinical characteristics of the HNSCC samples in training, validation, whole sets

	Training set (*n* = 300)	Validation set (*n* = 199)	Whole set (*n* = 499)
Gender (%)			
Male	223 (74.3%)	143 (71.9%)	366 (73.3%)
Female	77 (25.7)	56 (28.1%)	133 (26.7%)
Age (median, range)	60 (19–900)	61 (26–87)	61 (19–90)
Survival status			
OS days (media, range)	547(1–5252)	580 (14–6417)	558 (1–6417)
OS state (alive (%)/dead (%))	178 (59.3%)/122 (40.7%)	126 (63.3%)/73 (36.7%)	304 (60.9%)/195 (39.1%)
Grade (%)			
Grade1	44 (14.7%)	17 (8.6%)	61 (12.2%)
Grade2	180 (60.0%)	118 (59.3%)	298 (59.7%)
Grade3	67 (22.3%)	52 (26.1%)	119 (23.9%)
Grade4	2 (0.7%)	0 (0.0%)	2 (0.4%)
Unknown	7 (2.3%)	12 (6%)	19 (3.8%)
Stage (%)			
I	14 (4.7%)	11 (5.5%)	25 (5.0%)
II	44 (14.7%)	25 (12.6%)	69 (13.8%)
III	40 (13.3%)	38 (19.1%)	78 (15.7%)
IV	160 (53.3%)	99 (49.7%)	259 (51.9%)
Unknown	42 (14.0%)	26 (13.1%)	68 (13.6%)
*T* (%)			
0	1 (0.3%)	0 (0.0%)	1 (0.2%)
1	27 (9%)	18 (9.0%)	45 (9.0%)
2	72 (24.0%)	59 (29.6%)	131 (26.3%)
3	58 (19.3%)	38 (19.1%)	96 (19.2%)
4	110 (36.7%)	61 (30.7%)	171 (34.3%)
Unknown	32 (10.7%)	23 (11.6%)	55 (11.0%)
*N* (%)			
0	104 (34.7%)	66 (33.2%)	170 (34.1%)
1	36 (12%)	29 (14.6%)	65 (13.0%)
2	103 (34.3%)	61 (30.6%)	164 (32.9%)
3	3 (1%)	4 (2.0%)	7 (1.4%)
Unknown	54 (18%)	39 (19.6%)	93 (18.6%)

**Table 3:** 20 PR-irlncRNAs pairs and corresponding coefficients used to construct prognostic model

Gene	Coef
AL390719.2|AL133243.2	−0.04666
AC096992.2|HOXC-AS1	−0.2016
AC007038.1|AC084018.1	0.114178
ZNF687-AS1|AL354733.3	0.221731
TMPO-AS1|ATP1B3-AS1	0.288711
C5orf66-AS1|KDM4A-AS1	−0.07542
C5orf66-AS1|AP000251.1	−0.12957
AC106820.3|KDM4A-AS1	−0.62719
PTOV1-AS2|SNHG25	−0.16137
PTOV1-AS2|AL132712.1	−0.00603
PTOV1-AS2|LINC00205	−0.15718
AC132192.2|AC004148.1	0.042828
AC008735.2|SNHG25	−0.12269
LINC01063|AC116914.2	0.151718
RUSC1-AS1|AC004687.1	0.262677
LINC00944|AC004687.1	0.170279
CHKB-DT|AL365330.1	0.141328
MIR924HG|MIR9-3HG	0.177316
AL360181.2|AC098487.1	−0.01644
AL365330.1|MIR9-3HG	0.292315

### Predictive capability test of prognostic model

**Fig. 3:**
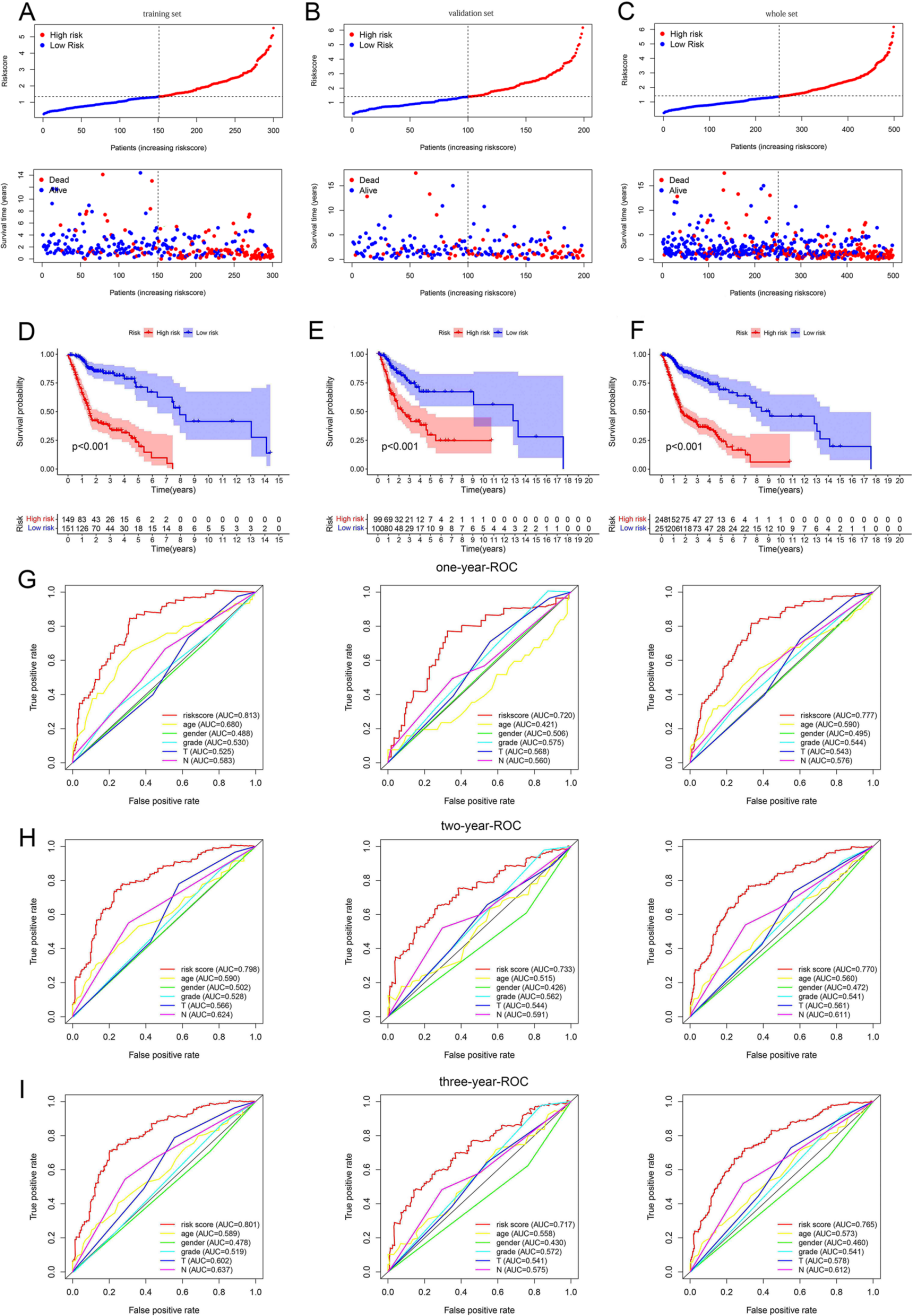
We used risk plots and survival point map, survival curves, and 1-, 2-, and 3-year multi-factor ROC curve to assess the performance of the prognostic model, which was respectively established by the data in the TCGA training set, test set, and the whole set. **A–C** Risk plots and survival point map.** D–F** Survival curves. **G** 1-year multi-factor ROC curves. **H** 2-year multi-factor ROC curves. **I** 3-year multi-factor ROC curves

The distribution of risk score, survival time, and status of three sets was shown in Fig. 3A–C, which indicated that patients with higher risk scores had shorter OS and worse survival conditions. In all sets, we observed a significantly higher survival probability in the low-risk patients compared to the high-risk patients (Fig. 3D–F), which showed the ability of our prognostic model to identify high-risk patients based on their survival conditions. Figure 3G–I illustrated that the area under the curve (AUC) values of risk scores for all sets at 1, 2, and 3 years exceeded 0.7, indicating the higher accuracy of our model’s prognosis prediction. In addition, we also found that the AUC values of the risk score at 1, 2, and 3 years were all higher than other clinical factors, which represented that our model had the optimal effect. The results of the univariate Cox regression analysis in Table 4 revealed the significant correlation between survival time/state and the risk score of the three sets (*p* < 0.05). After adjusting other clinical confounding factors by multivariate Cox regression analysis, the risk scores of the three sets still remained significantly correlated with survival (*p* < 0.05), thereby indicating that the risk score could serve as an independent predictor of prognosis.

**Table 4:** The results of univariate and multivariate Cox regression analysis include clinical factors and risk scores in three sets

Variables	Univariate cox		*P* ^1^	Multivariate cox		*P* ^2^
	HR	95%CI		HR	95%CI	
Training set						
Age	1.03	1.02-1.05	*<0.001*	1.03	1.02-1.05	*<0.001*
Gender	0.90	0.58-1.38	0.630	0.90	0.57-1.42	0.642
Grade	0.99	0.74-1.33	0.955	1.05	0.74-1.48	0.795
T	1.29	1.05-1.58	*0.015*	1.16	0.92-1.47	0.215
N	1.48	1.20-1.83	*<0.001*	1.45	1.11-1.90	*0.006*
Risk score	2.25	1.89-2.68	*<0.001*	2.12	1.76-2.56	*<0.001*
Test set						
Age	1.01	0.99-1.03	0.483	0.99	0.97-1.02	0.582
Gender	0.59	0.35-0.99	*0.045*	0.60	0.34-1.06	0.081
Grade	1.42	0.94-2.13	0.095	1.43	0.92-2.22	0.109
T	1.21	0.93-1.55	0.149	0.98	0.73-1.30	0.878
N	1.30	0.98-1.65	0.075	1.22	0.92-1.61	0.168
Risk score	1.58	1.33-1.87	*<0.001*	1.53	1.28-1.83	*<0.001*
Whole set						
Age	1.02	1.01-1.04	*<0.001*	1.02	1.00-1.03	*0.016*
Gender	0.76	0.55-1.06	0.101	0.93	0.65-1.33	0.691
Grade	1.12	0.88-1.42	0.357	1.11	0.86-1.44	0.425
T	1.27	1.08-1.49	*0.004*	1.05	0.88-1.26	0.574
N	1.38	1.17-1.63	*<0.001*	1.31	1.10-1.57	*0.003*
Risk score	1.78	1.59-1.99	*<0.001*	1.66	1.48-1.87	*<0.001*

Italics indicates that the results are statistically significant

*HR* Hazard ratio, *CI* Confidence interval

### Relationship between risk score and clinical characteristics

**Fig. 4:**
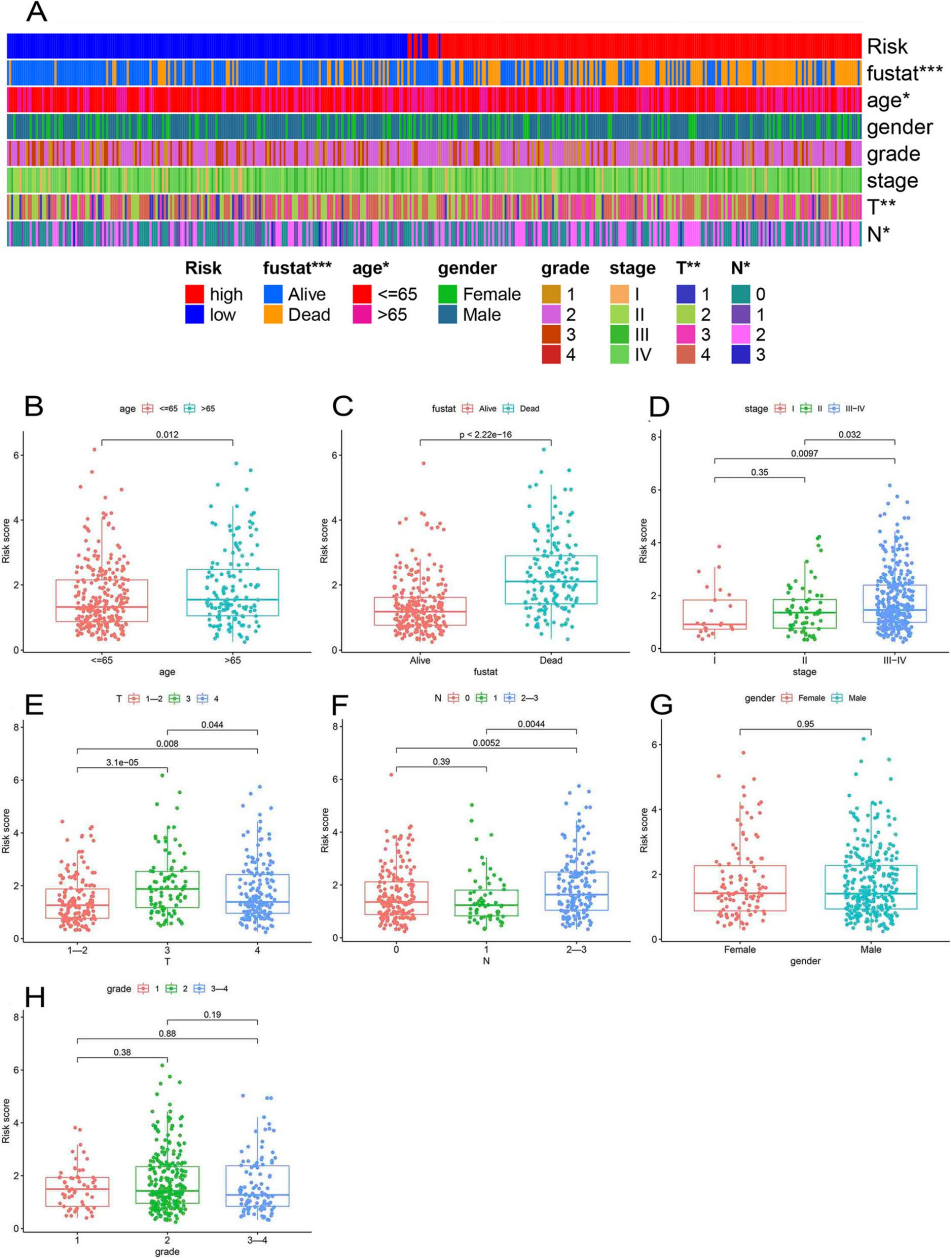
The relation between different subgroups of each clinical feature and risk score. **A** The distribution of different subtypes of each clinical feature for each sample with the increase of risk score. **B–H** Risk score differences between patients with different subtypes in different clinicopathological features

The distribution of clinical features of all samples with the increase of risk score was shown in Fig. 4A, indicating a significant association between fustat (*p* < 0.001), age (*p* < 0.05), *T* (*p* < 0.01), and *N* (*p* < 0.05) and the risk score calculated by the prognostic model. Furthermore, higher mortality observed in samples with the advanced T stage and N stage was more concentrated in the high-risk group. It was noteworthy that many clinically valuable results were found in the further difference analysis of the relationship between risk score and different clinical characteristics groups (Fig. 4B–H). Higher risk scores existed in older than 65 and dead samples (Fig. 4B–C). Samples in stage III–IV owned higher risk scores compared to stage I and II (Fig. 4D). Figure 4E demonstrated the positive relationship between the risk score and T grade. An analogous result was also discovered in the N stage (Fig. 4F). Further survival analysis showed that the prognostic model had a superb ability for OS across each of the other subgroups with distinct clinical characteristics (Fig. S2A-G, 5I–P), except the stage I subgroup (Fig. S2H). Additionally, within these subgroups, samples in the high-risk group exhibited inferior OS (*p* < 0.05).

**Fig. 5:**
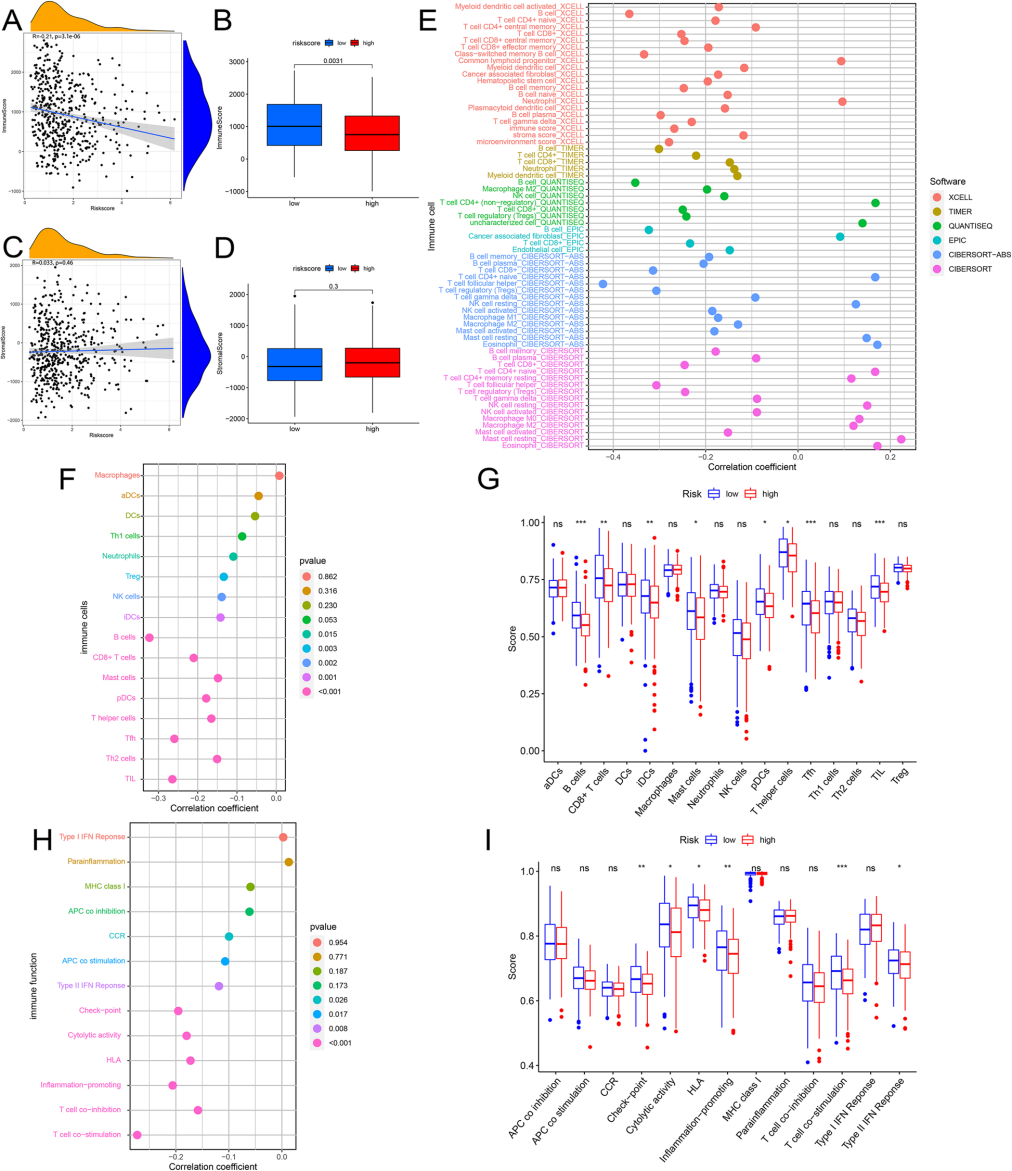
The association analysis between risk score and immune cells scores, stromal cells score, immune cell content calculated by six most advanced algorithms, 16 immune infiltrating cells and 13 immune functions calculated by ssGSEA and the comparison of different parameters between different risk groups, including immune cells score, stromal cells score, 16 immune infiltrating cells, and 13 immune functions. **A, B** Immune cells score. **C, D** Stromal cells score. **E** Immune cell content calculated by the six most advanced algorithms. **F, G** 16 immune infiltrating cells. **H, I** 13 immune functions. The symbol above the histogram shows the significance of the difference. *p < 0.05; **p < 0.01; ***p < 0.001; ns, no significance

### Characteristics of immune microenvironment in HNSCC

The Spearman correlation results implied the negative correlation between the risk score and the immune cell score (*p* < 0.001) (Fig. 5A). Higher immune cell scores were discovered in patients in the low-risk group (*p* < 0.01) (Fig. 5B), which further confirmed the accuracy of the correlation results. Unfortunately, no significant results were found between stromal cell score and risk score (Fig. 5C–D). To enhance the accuracy of the data, we used the immune infiltrating cell contents using six most advanced algorithms and ssGSEA for calculation in this part of the comprehensive analysis. Following the Spearman correlation analysis, B cell, T cell CD8+, myeloid dendritic cell, B cell memory, B cell plasma, T cell gamma delta, T cell follicular helper (Tfh) cell, Treg cell, mast cell activated, and natural killer (NK) cell activated were observed to have the negative correlation with the risk score, while mast cell resting and eosinophil had the positive correlation with the risk score. The bubble chart displayed all the results of the correlation analysis(Fig. 5E–F). In the further Mann–Whitney *U* test, significant differences in the content of almost all cells between the high-risk and low-risk groups were found (Fig. 5G). Besides, the risk score was found to have a negative correlation with the scores of C-C Chemokine receptors (CCR), antigen‐presenting cell (APC) co-stimulation, type II IFN response, checkpoint, cytolytic activity, human leukocyte antigen (HLA), inflammation-promoting, T cell co-stimulation, and T cell co-inhibition (*p* < 0.05) (Fig. 5H). Among them, check-point, cytolytic activity, HLA, inflammation-promoting, T cell co-stimulation, and type II interferons (IFN) response in the low-risk group were observed to own higher scores (*P* < 0.05) (Fig. 5I). Figure S3A-C further supports the relevant results by the computation of other methods.

### Mutations associated with prognostic model

**Fig. 6:**
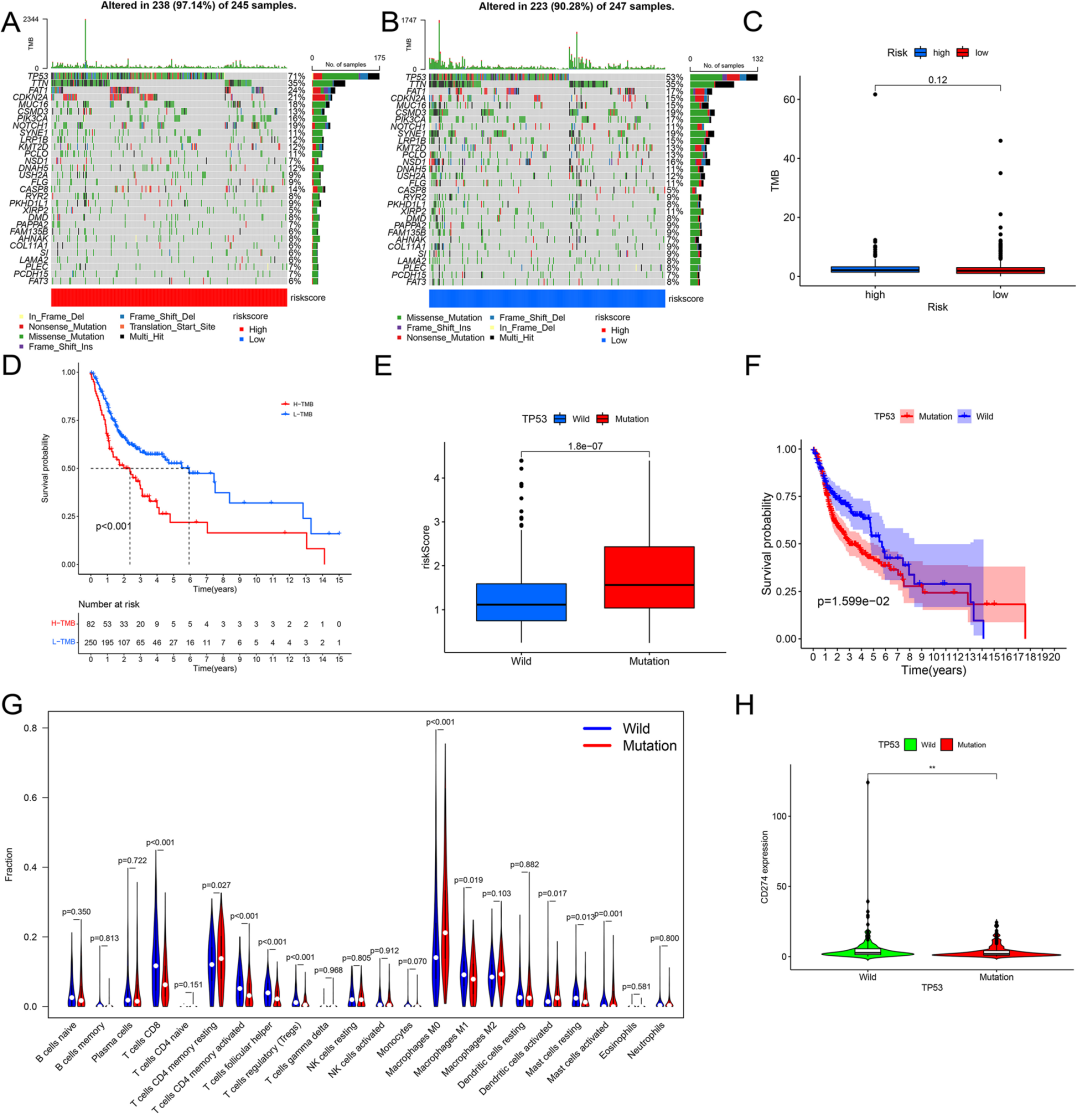
Mutation analysis. **A, B** Waterfall charts reflect the gene mutations of patients in the high-risk and low-risk groups. **C** The contrast of TMB content between the high-risk group and low-risk group. **D** The KM survival correlation analysis shows the difference between the high TMB and the low TMB groups. **E** The display of the risk score differences between the TP53 mutant group and TP53 wild group. **F** The KM survival analysis reveals the difference in survival probability between the TP53 mutation group and TP53 wild groups. **G** The fraction differences between the TP53 mutant group and TP53 wild group in 22 immune cells. **H** The difference in the expression level of PDL1 between the high-risk and low-risk groups

We demonstrated the overviews of mutations in the top 30 most common genes for 238 samples from the high-risk group and 223 from the low-risk group, respectively (Fig. 6A–B). Despite no difference in TMB between the two groups, a worse prognosis was still observed in the high TMB group (Fig. 6C–D). As anticipated, a higher risk score (*p* < 0.001) (Fig. 6E) and a worse prognosis (*p* <0.05) (Fig. 6F) were discovered in the patients with TP53 mutations. More than that, we observed significantly lower content of T cells CD8, T cells CD4 memory activated, Tfh, Treg, Macrophages M1, Mast cells resting, and Mast cells activation of patients in the TP53 mutation group (*p* < 0.05) (Fig. 6G). Conversely, the content of T cells CD4 memory resting, macrophages M0, and dendritic cells resting was strikingly higher in the TP53 mutation group (Fig. 6G). At the same time, the expression of PDL1 (CD274) was significantly lower in the TP53 mutant group (Fig. 6H).

### Correlation between prognostic model and ICIs/m6A/multidrug resistance-related genes’ expression

We evaluated the correlation between 46 ICIs-related genes and risk scores (Fig. S4A-B). The expression of TNFSF9, CD44, and CD276 had a significant positive correlation with the risk score, while the other 30 ICIs-related genes showed a negative correlation with risk scores, except for CD80, PDCD1LG2, HHLA2, CD70, CD86, NRP1, ICOSLG, CD274, TNFSF18, CD40, IDO1, HAVCR2, and BTNL2. Except for TNFSF9 and TNFRSF9, the differential expression of other genes with significant correlation in Fig. S4A was again confirmed in different risk scores (Fig. S4B). In addition to CD44 and CD276, the other 29 ICIs-related genes in the low-risk group had higher expression levels. Besides, we also found that the risk score had a negative correlation with the expression level of YTHDC1 and YTHDC2 while a positive correlation with HNRNPC (Fig. S4C). Aside from HNRNPC, we tested the significant correlation results of YTHDC1 and YTHDC2 in the difference analysis (Fig. S4D). In addition, we discovered strikingly higher expression levels of RBM15, YTHDC1, YTHDC2, METTL14, WTAP, and METTL3 in the low-risk group. Although there was no significant correlation between the expression of the resistance gene MRP1 (ABCC1) and the risk score, higher MRP1 expression was still observed in the low-risk group (Fig. S4E-F). Meanwhile, no significant results were seen in the correspondence analysis of MRP3 (ABCC3) (Fig. S4G-H).

### Prognostic model associated with immunotherapy effect and chemotherapy sensitivity

**Fig. 7:**
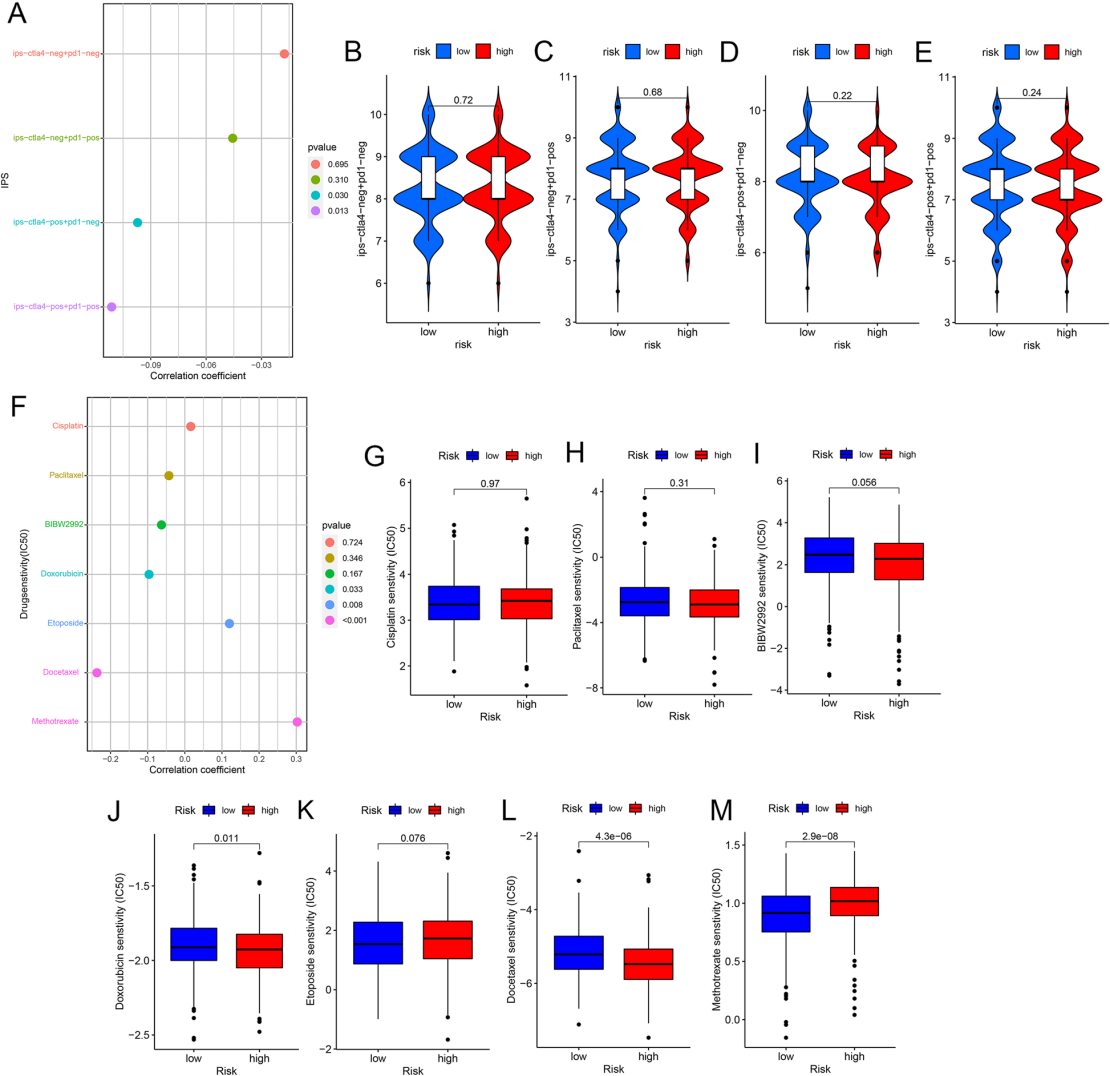
The relevance analysis between risk score and four kinds of IPSs and sensitivity of the seven chemotherapy drugs and their comparison between low-risk and high-risk groups. **A–E** Four kinds of IPSs. **F–M** Sensitivity test scores of 7 chemotherapeutic drugs

The IPS evaluated the curative effect of the corresponding ICIs administered to patients. Patients with higher IPS exhibited a superior response to ICIs [49]. Although there is no practical evidence for further difference analysis (Fig. 7B–E), we still found that IPS-CTLA4-POS+PD1-NEG and IPS-CTLA4-POS+PD1-POS had a negative correlation with the risk score (Fig. 7A). We also predicted the IC50s of 7 chemotherapeutic agents used for HNSCC treatment, including cisplatin, paclitaxel, BIBW2992, doxorubicin, etoposide, docetaxel, and methotrexate. The IC50 of doxorubicin and docetaxel was observed to negatively correlate with a risk score, while etoposide and methotrexate had a positive one (Fig. 7F). Except for etoposide, the correlation results of the other three chemotherapeutic agents were supported by further difference analysis (Fig. 7J, L–M). Figure 7G–M shows the results of the differential analysis for these seven drugs. It was concluded that our model might hold potential in predicting the therapeutic efficacy of the corresponding immunotherapy and chemotherapy.

### Building and evaluation of Nomogram

**Fig. 8:**
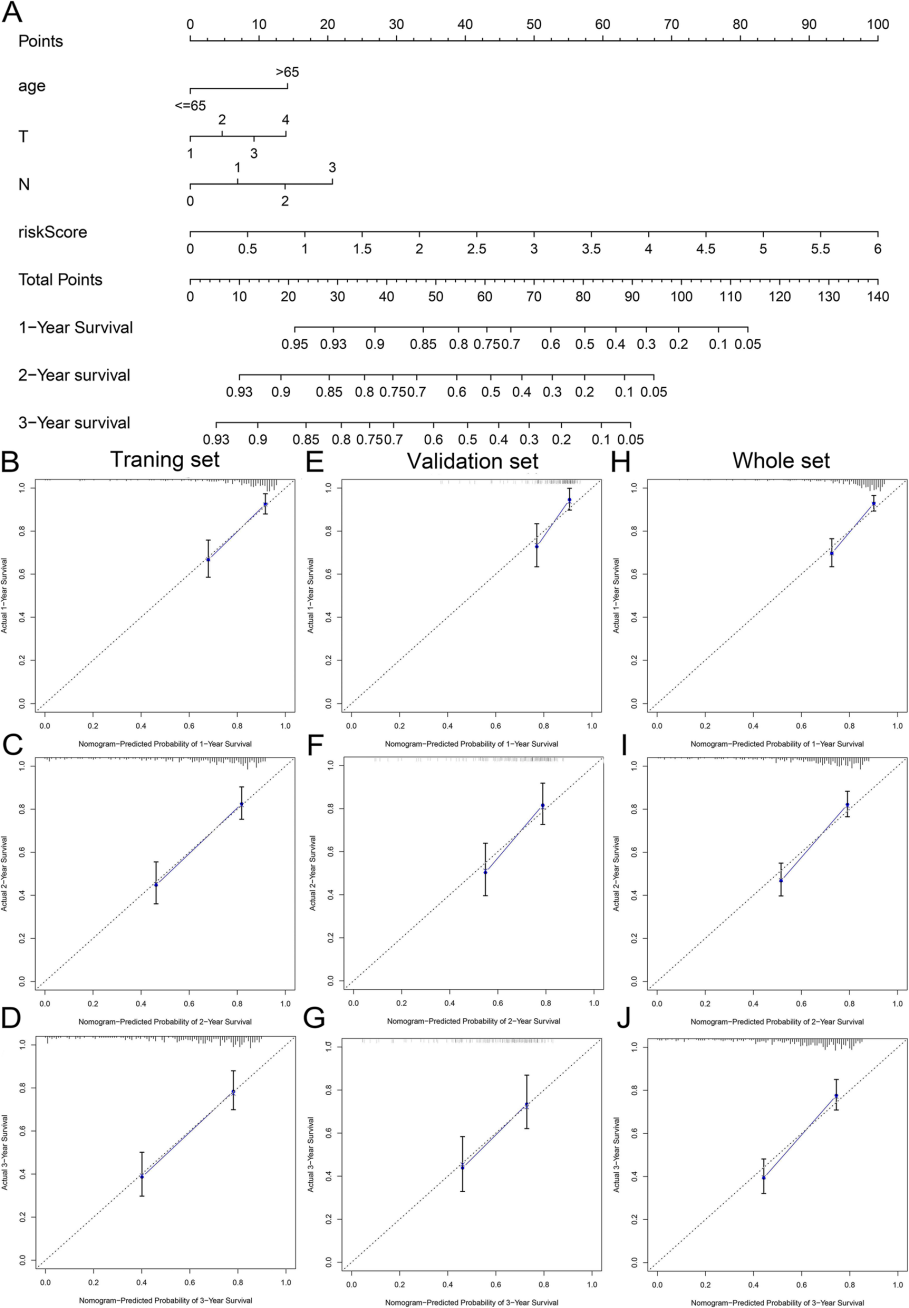
Establishment and verification of Nomogram. **A** The Nomogram with the effects of predicting the 1-, 2-, and 3-year survival probability of HNSCC patients. 1-, 2-, and 3-year internal calibration curves are displayed respectively. **B–D** Based on the training set. **E–G** Based on the test set. **H–J** Based on the whole set

Age, T stage, N stage, and risk score were subsumed into multivariate Cox regression model, and the relevant data from the training set samples were used to draw the Nomogram (Fig. 8A). After applying the calibration curve, it was confirmed that the Nomogram exhibited good consistency in predicting the actual OS at 1, 2, and 3 years across three sets (Fig. 8B–J). The multi-factor ROC also supported the effectiveness of the Nomogram in predicting survival rate, with our Nomogram owning a better ability to predict survival (AUC >0.7) and excelling in all factors predicting survival across all sets (Fig. S5).

### Gene set enrichment analysis

Two hundred thirty differential genes were identified between the high- and low-risk groups. Biological processes (BP), cellular component (CC), and molecular functions (MF) significantly related to these genes were enriched and displayed in Fig. S6A. Enriched BPs have been observed to be all related to immunity, such as humoral immune response mediated by circulating immunoglobulin, complement activation, classical pathway, complement activation, immunoglobulin-mediated immune response, B cell-mediated immunity, humoral immune response, lymphocyte-mediated immunity, adaptive immune response based on somatic recombination of immune receptors built from immunoglobulin superfamily domains, immune response-activating, cell surface receptor signaling pathway, and immune response-activating signal transduction. In addition, immune-related functions such as immunoglobulin complex, immunoglobulin complex, circulating, antigen binding, and immunoglobulin receptor binding are observed in enriched CCs and MFs. Unfortunately, immune-related pathways have not been enriched, replaced by nicotine addiction, primary immunodeficiency, linoleic acid metabolism, hematopoietic cell lineage, IL-17 signaling pathway, mineral absorption, staphylococcus aureus infection, drug metabolism-cytochrome P450, estrogen signaling pathway, cell adhesion molecules, and ferroptosis (Fig. S6B).

### Validation of abnormal expression of modeled genes in HNSCC cells

**Fig. 9:**
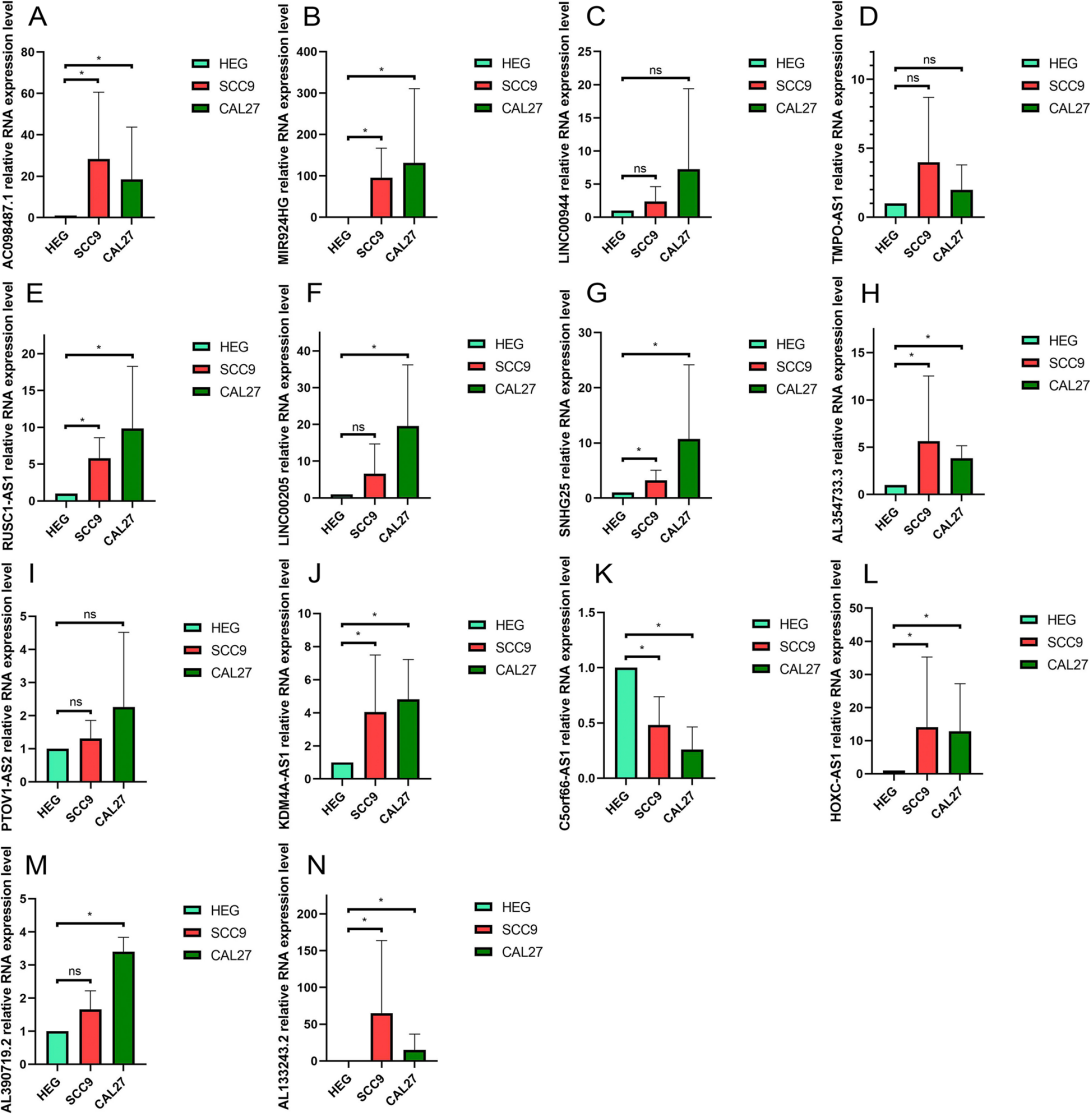
Validation of abnormal expression of modeled genes in HNSCC cells. **A** AC098487.1. **B** MIR924HG. **C** LINC00944. **D** TMPO-AS1. **E** RUSC1-AS1. **F** LINC00205. **G** SNHG25. **H** AL354733.3. **I** PTOV1-AS2. **J** KDM4A-AS1. **K** C5orf66-AS1. **L** HOXC-AS. **M** AL390719.2. **N** AL133243.2

In OSCC cells (scc9 or cal27), the relative RNA expression levels of AC098487.1, MIR924HG, RUSC1-AS1, LINC00205, SNHG25, KDM4A-AS1, AL354733.3, HOXC-AS1, AL390719.2, and AL133243.2 were higher (Fig. 9A–B, E–H, J, and L–N). In addition, we also observed significantly lower relative RNA expression levels of C5orf66-AS1 in OSCC cells (scc9 or cal27, Fig. 9K). These results were consistent with those obtained by bioinformatics analysis. Unfortunately, we did not observe significantly abnormal relative RNA expression levels of LINC00944, PTOV1-AS2, and TMPO-AS1 in OSCC cells (Fig. 9C–D and I).

## Discussion

Currently, most of these predictive signatures were combinations of single lncRNAs [50, 51]. However, in contrast, the dual biomarker combination has been shown to outperform a single marker in terms of the accuracy of cancer diagnostic models [32]. Inspired by the strategy of matching immune-related genes, we paired the DE-irlncRNAs into lncRNA pairs that was not affected by the expression level. We constructed a prognostic model based on 20 PR-DE-irlncRNAs pairs. First, we selected the PR-DE-irlncRNAs pairs that were effectively matched based on the data obtained from the TCGA database and subsequently developed an effectively prognostic model for HNSCC patients. The predictive value of the model was then verified through a variety of methods. Moreover, our model was closely related to ICIs/m6A/multidrug resistance-related genes’ expression, with excellent clinical applicability. Finally, our analysis revealed that immune cells, immune function, and TP53 mutations may be implicated in the progress of HNSCC.

As a current research hotspot, irlncRNAs have been used in the signatures of various cancers. AC007038.1|AC084018.1, ZNF687−AS1|AL354733.3, TMPO−AS1|ATP1B3−AS1, AC132192.2|AC004148.1, LINC01063|AC116914.2, RUSC1−AS1|AC004687.1, LINC00944|AC004687.1, CHKB−DT|AL365330.1, MIR924HG|MIR9−3HG, and AL365330.1|MIR9−3HG in model played negative significant roles, while AL390719.2|AL133243.2, AC096992.2|HOXC−AS1, C5orf66−AS1|KDM4A−AS1, C5orf66−AS1|AP000251.1, AC106820.3|KDM4A−AS1, PTOV1−AS2|SNHG25, PTOV1−AS2|AL132712.1, PTOV1−AS2|LINC00205, AC008735.2|SNHG25, and AL360181.2|AC098487.1 played positive roles in HNSCC patients’ survival. The PR-DE-irlncRNAs used in this study to establish a prognostic model have also been determined to possess a brilliant predictive value for the prognosis of patients in other cancers.

Xu et al. identified AL390719.2 as one of the key prognostic lncRNAs for both 10- and 5-year survival rates in colorectal cancer [52]. C5orf66-AS1 prevents oral squamous cell carcinoma by inhibiting cell growth and metastasis [53] and has been verified as a biomarker for various cancers [54, 55]. Studies have demonstrated that AL354733.3 exhibits a positive correlation with autophagy genes and can serve as an independent prognostic indicator for OSCC patients [56]. TMPO‐AS1 regulates the proliferation and migration of triple‐negative breast cancer cells by modulating transforming growth factor‐β and E2F signaling pathways [57]. Moreover, TMPO-AS1 has the potential to enhance LCN2 transcriptional activity by binding to transcription factor E2F6, thus stimulating ovarian cancer progression [58]. Feng et al. revealed that AC116914.2 is significantly related to the expression of PD-L1 in primary head and neck squamous cell carcinoma [59]. Cheng et al. stated that LINC01063 is a risk-related autophagy-related lncRNA with a poor prognosis in colorectal cancer [60], which was confirmed again in the study of Zhou et al. [61]. Ye et al. reported that AC004687.1 is significantly related to recurrence-free survival of hepatocellular carcinoma patients [62]. It was shown that RUSC1-AS1 correlated with the prognosis of various cancers [63–65]. For instance, it activates NOTCH signaling via the hsa-miR-7-5p/NOTCH3 axis, promoting the proliferation and reducing the apoptosis of HCC cells [66]. Moreover, RUSC1-AS1 promotes the aggressiveness of cervical cancer in vitro and in vivo by upregulating miR-744-Bcl-2 axis output [67]. De Santiago et al. showed that LINC00944 is in response to ADAR1 up- and downregulation in breast cancer cells, and the low expression of LINC00944 is correlated to poor prognosis in breast cancer patients [68]. MIR9-3HG was identified as a key lncRNA with diagnostic and prognostic value for HNSCC [69] and liver hepatocellular carcinoma [70]. LncRNA HOXC-AS1 promotes nasopharyngeal carcinoma progression by sponging miR-4651 to upregulate FOXO6 [71]. Deng et al. identified signature lncRNAs that could serve as predictors of the OS rate of hepatocellular carcinoma [72]. PTOV1-AS2 was used to construct a tp53-associated nomogram to predict the OS in patients with pancreatic cancer [73]. High expression levels of the LINC00205 correlate with a better OS in pancreatic cancer [74]. The study of Yang et al. revealed that OS was significantly shortened in the SNHG25 high expression group and significantly upregulated in clear cell renal cell carcinoma (ccRCC) tissues [75]. Another study identified AL360181.2 and AC008735.2 as potential prognostic markers to construct a model for predicting the prognosis of ccRCC patients [76].

This study showed a significant correlation between the patient’s risk score and prognosis, with different survival probabilities observed between different risk subgroups. Furthermore, the clinical stratification analysis showed that risk score still maintained the ability to distinguish the prognosis of patients with high- and low-risk across different subgroups. These all highlighted the accuracy and optimality of the predictive ability of the prognostic model.

We analyzed 230 differential genes between high-risk and low-risk populations. We discovered that the enriched molecular functions were related to immunity, and immune-related functions were also observed in the enriched cellular components and molecular functions. This indicated a close relationship of our model with immunity. Therefore, we further explored the relationship between tumor immunity and our risk model from the perspective of the immune microenvironment, including immune infiltrating cells and immune function. Immune infiltrating cells in tumors played an essential role in the occurrence and development of tumors, ultimately impacting patient prognosis [77]. Therefore, understanding tumor immune infiltrating cells could explore the prognosis of tumor patients and the new direction of HNSCC treatment in the future. We observed that B cell, T cell CD8+, myeloid dendritic cell, B cell memory, B cell plasma, T cell gamma delta, Tfh cell, Treg cell, mast cell activated, and NK cell activated negatively correlated with the risk score, while mast cell resting and eosinophil were positively correlated. Even further, the significant differences in cell content between high- and low-risk groups supported the credibility of the results. B cells played an important role in anti-tumor immunity. The presence of NK cells and NK T cells in most solid tumors often meant a good prognosis [78].

In addition, the scores of CCR, APC co-stimulation [79], type II IFN response [80], checkpoint, cytolytic activity, HLA [81], inflammation-promoting, T cell co-stimulation, and T cell co-inhibition were observed to be negatively correlated with risk score (*p* < 0.05). Among them, higher scores of checkpoint, cytolytic activity, HLA, inflammation-promoting, T cell co-stimulation, and type II IFN response were observed in the low-risk group (*p* < 0.05). CCR 5 could recruit MDSC to tumors closely related to tumor immunity [82]. Type II IFN-γ has the potential to induce tumor cell apoptosis and regulate cancer immune activity [80]. Previous studies have demonstrated that primary colorectal cancer and corresponding metastases usually exhibit downregulation or loss of HLA-I expression [81]. Tumor may drive the immune escape by changes in HLA expression (or by other means) [83], which might be developed into auxiliary tumor markers in the future. These conclusions implied that a multitude of immune infiltrating cells in the low-risk group may participate in the anti-HNSCC response through a series of immune functions, ultimately leading to improved patient prognoses.

Among the mutations in HNSCC, TP53 mutation was found to be the most common mutation in both high-risk and low-risk groups. It was well-established that the number of p53-regulated lncRNA increased rapidly, indicating their widespread involvement downstream of p53 activation [84]. Transcription factor p53 was a most prominent human tumor suppressor that played an essential role in cellular responses to DNA damage stimuli [85]. As anticipated, patients in the TP53 mutation group had higher risk scores and worse prognoses. Moreover, we also found that the contents of T cell CD8, T cell CD4 memory activation, Tfh cell, Treg cell, macrophage M1, mast cell rest, and mast cell activation were significantly lower in the TP53 mutation group. Conversely, there was a significant increase in the contents of T cell CD4 memory rest, macrophage M0, and dendritic cell rest within this same group. In head and neck cancers, the presence of TP53 mutations was associated with lower estimates of various immune infiltrating cells, such as T, B, and NK cells [86]. Increased levels of M0 macrophages were associated with poor clinical outcomes in lung adenocarcinoma [87]. Furthermore, relevant studies have shown that M0 macrophages promoted malignant progression and were affected by tumor development [87].

In addition, we found that the expression of PDL1 (CD274) decreased significantly in the TP53 mutation group, which may lead to an increase in the tumor and cancer stem cell phenotype in cholangiocarcinoma. At the same time, it was found that low CD274 had high tumor initiation potential [88]. At present, the PD-L1 signal contributes to human cancer immune escape, thereby blocking PD-L1 has been applied to clinical cancer treatment [89, 90]. The significant efficacy of PD-L1 blockers in cancer immunotherapy was expected to control cancer by regulating the expression of PD-L1 [91, 92], which has been shown to have a potential predictive effect in melanoma, non-small-cell lung cancer, renal cell carcinoma, prostate cancer, or colorectal cancer [93, 94]. These results suggested that in HNSCC, TP53 mutation may promote the progress of HNSCC by suppressing these immune cells and inhibiting anti-tumor immunity, ultimately leading to a poor prognosis. Furthermore, in HNSCC, patients with TP53 mutations may benefit less from PD-1 treatment. Nowadays, immunotherapy is an emerging strategy for anti-tumor therapy. Therefore, our study investigated the relationship between immune-related genes and the prognosis model. We found that the expression of CD44 and CD276 had a significantly positive correlation with a risk score. As a CSC marker of HNSCC, CD44 participates in the DNA damage response of G2/M phase arrest. Overexpression of CD44 provided relative protection for HNSCC cells against cell death response [95]. CD276, a member of the B7 family, was considered a factor that regulated antigen-specific T cell immune response through costimulatory and co-inhibitory receptors. The expression of CD276 was negatively correlated with the number of tumor-infiltrating CD8 + T cells, and the upregulated expression was related to the poor prognosis in esophageal cancer [96, 97]. In our study, patients in the high-risk group with more CD44 and CD276 expression exhibited a worse prognosis, which was consistent with the conclusions of these related studies.

More and more evidence showed that m6A RNA methylation played a crucial role in tumorigenesis. m6A modification of some genes may result in changes in mRNA behavior and expression, thus accelerating tumor development, whereas the lack of m6A modification of other genes might still lead to tumor progression [98]. We studied the relationship between m6A-related genes and prognostic models, from which the expression level of HNRNPC was positively correlated with the risk score. Overexpression of HNRNPC was found in the central regulators of colon rectum cancer cells and cancer progression-related genes [99]. The expression of HNRNPC might be related to poor prognosis, similar to our findings, providing valuable insights into the study m6A-related genes in HNSCC.

Our study observed that IPS-CTLA4 and IPS-PD1 + CTLA4 were negatively correlated with a risk score. Some experiments showed that in HNSCC, the scores of IPS with CTLA4 blocker, IPS with CTLA4, and PD1/PDL1/PdL2 blocker in the low-risk group were significantly higher than those in the high-risk group, which was consistent with our experimental results [100]. This meant that our prognostic model had a certain predictive value for the efficacy of patients receiving corresponding immunotherapy. Following calculation, we found that the IC50 of methotrexate was positively correlated with the risk score, while the IC50 of doxorubicin and docetaxel was negatively correlated with the risk score. From this, our prognostic model possesses a certain guiding significance for the use of chemotherapeutic drugs. Nomogram could provide personalized prognostic assessment for both surgeons and patients, serving as a reference for treatment planning [101]. The Nomogram drawn according to the relevant data from the training samples had an excellent ability to predict survival, which provided a new insight into the prognosis of HNSCC.

It is worth acknowledging that our research had certain shortcomings and limitations. Firstly, due to the lack of data sets containing complete lncRNA and mRNA transcription data in other shared databases, we only relied on a distinct data set from TCGA to build and validate our model, which may lead to randomness in the results. The lack of an external validation set posed a challenge to the reliability of model performance. To compensate for this limitation, three data sets obtained by randomly splitting the TCGA data set were used to thoroughly verify the model’s performance. In addition, it is noteworthy that the limited number of normal samples also poses challenges to the accuracy of differential analysis results. Furthermore, due to the large number of lncRNAs in the model and limited experimental conditions, it was challenging for us to perform qRT-PCR to verify the differential expression of these lncRNAs. However, we obtained many valuable conclusions through multi-perspective analysis, regarding both clinical application and the underlying mechanism of HNSCC progress. Nevertheless, these conclusions need to be further verified in subsequent experimental studies.

## Conclusions

The prognosis model included a total of 20 PR-DE-irlncRNAs pairs. Various methods have verified that the risk score calculated from 20 pairs of PR-DE-irlncRNAs has an excellent prognostic capability to predict the prognosis of patients with HNSCC. The prognostic model also performs well in the clinical risk classification and treatment guidance of HNSCC patients.

### Supplementary Information

**Additional file 1:** **Fig. S1.** The minimum 10-fold cross-validation determined the optimal penalty parameter (λ).

**Additional file 2:** **Fig. S2.** Survival correlation analysis in different subtypes of each clinical feature. The changes of survival probability with time in different subtypes of different clinical characteristics in high-risk and low-risk groups.

**Additional file 3:** **Fig. S3.** The six most advanced algorithms calculate the comparison between high and low-risk groups in different immune cell contents. (A) CIBERSORT and ABS, (B) XCELL, (C) TIMER, QUANTISEQ, and EPIC.

**Additional file 4:** **Fig. S4.** The relevance analysis between risk score and ICIs-related genes/m6a-related / multidrug resistance genes' expression level and the comparison of these parameters in different risk groups. (A, B) The expression level of ICIs-related genes. (C, D) The expression level of m6a-related genes. **(**E, F) The expression level of ABCC1. **(**G, H) The expression level of ABCC3.

**Additional file 5:** **Fig. S5.** The multi-factor ROC curves were used to confirm the Nomogram with the optimal predictive performance. 1-, 2-, and 3-years of multi-factor ROC curves based on three sets: (A-C) The training set. (D-F) The test set. (G-I) The whole set.

**Additional file 6:** **Fig. S6.** Results of GO and KEGG enrichment analysis. (A) BPs, CCs, and MFs that are strikingly enriched by GO. (B) Pathways are strikingly enriched by KEGG. Different colors of circles and rectangles represent different significances. The size of the circle corresponds to the different ratios of DEGs enriched by each function to the total number of DEGs.

## Data Availability

The datasets analyzed in this study came from databases shared publicly. Data can be obtained from GDSC https://www.cancerrxgene.org/, ImmPort database https://www.immport.org/shared/home, TCGA https://cancergenome.nih.gov/, TCIA https://tcia.at/home.

## Abbreviations

APC: Antigen-presenting cell
AUC: Area under the curve
BP: Biological processes
CC: Cellular component
CCR: C-C Chemokine receptors
ccRCC: Clear cell renal cell carcinoma
CSCs: Cancer stem cell-like cells
DEGs: Differential expressed genes
DE-irlncRNAs: Differentially expressed immune-related lncRNAs
GDSC: Cancer Drug Sensitivity Genomics
GO: Gene Ontology
GSEA: Gene set enrichment analysis
HCC: Hepatocellular carcinoma
HLA: Human leukocyte antigen
HNSCC: Head and neck squamous cell carcinoma
IC-50: Half-inhibitory concentration
ICIs: Immune checkpoint inhibitors
IFN: Interferons
IPS: Immunophenotypic score
IRGs: Immune-related genes
KEGG: Kyoto Encyclopedia of Genes and Genomes
K-M: Kaplan–Meier
lncRNA: Long-chain non-coding RNA
MF: Molecular functions
NK cell: Natural killer cell
OS: Overall survival
PR-DE-irlncRNAs: Prognostic-related DE-irlncRNAs
ROC: Receiver Operating Characteristic
ssGSEA: Single-sample gene set enrichment analysis
TCGA: The Cancer Genome Atlas database
TCIA: The Atlas of Cancer Immunity
Tfh cell: T follicular helper cell
TIME: Tumor immune microenvironment
TMB: Tumor mutation burden
Treg: Regulatory T
