# Supplementary figures and images for: A new marker constructed from immune-related lncRNA pairs can be used to predict clinical treatment effects and prognosis: in-depth exploration of underlying mechanisms in HNSCC
Source: World J Surg Oncol. 2023 Aug 17;21:250. doi: 10.1186/s12957-023-03066-x (PMC10433616; doi:10.1186/s12957-023-03066-x)

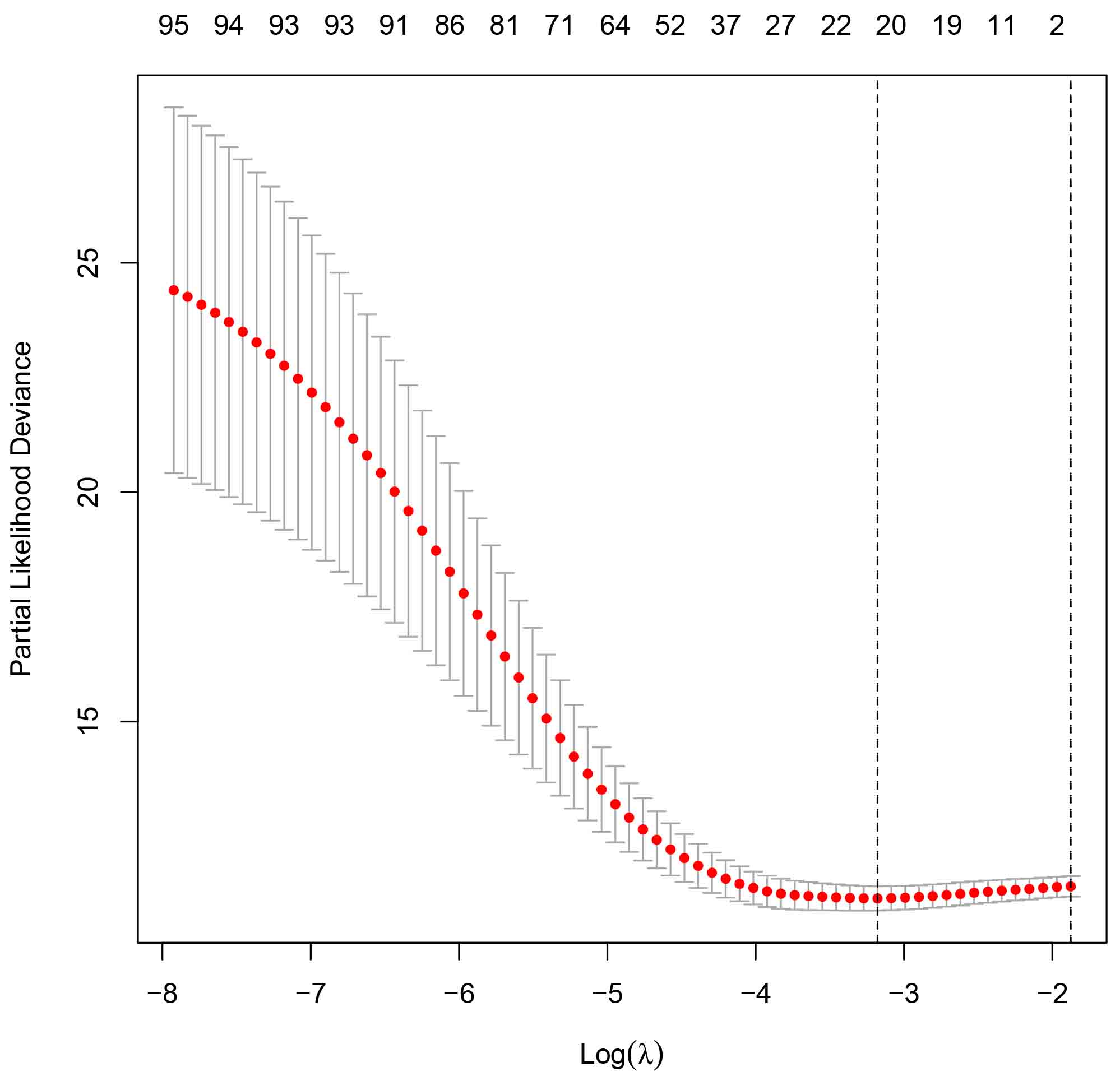

Supplement: Supplementary file 1 — Additional file 1: Fig. S1. The minimum 10-fold cross-validation determined the optimal penalty parameter (λ). [file 12957_2023_3066_MOESM1_ESM.jpg]

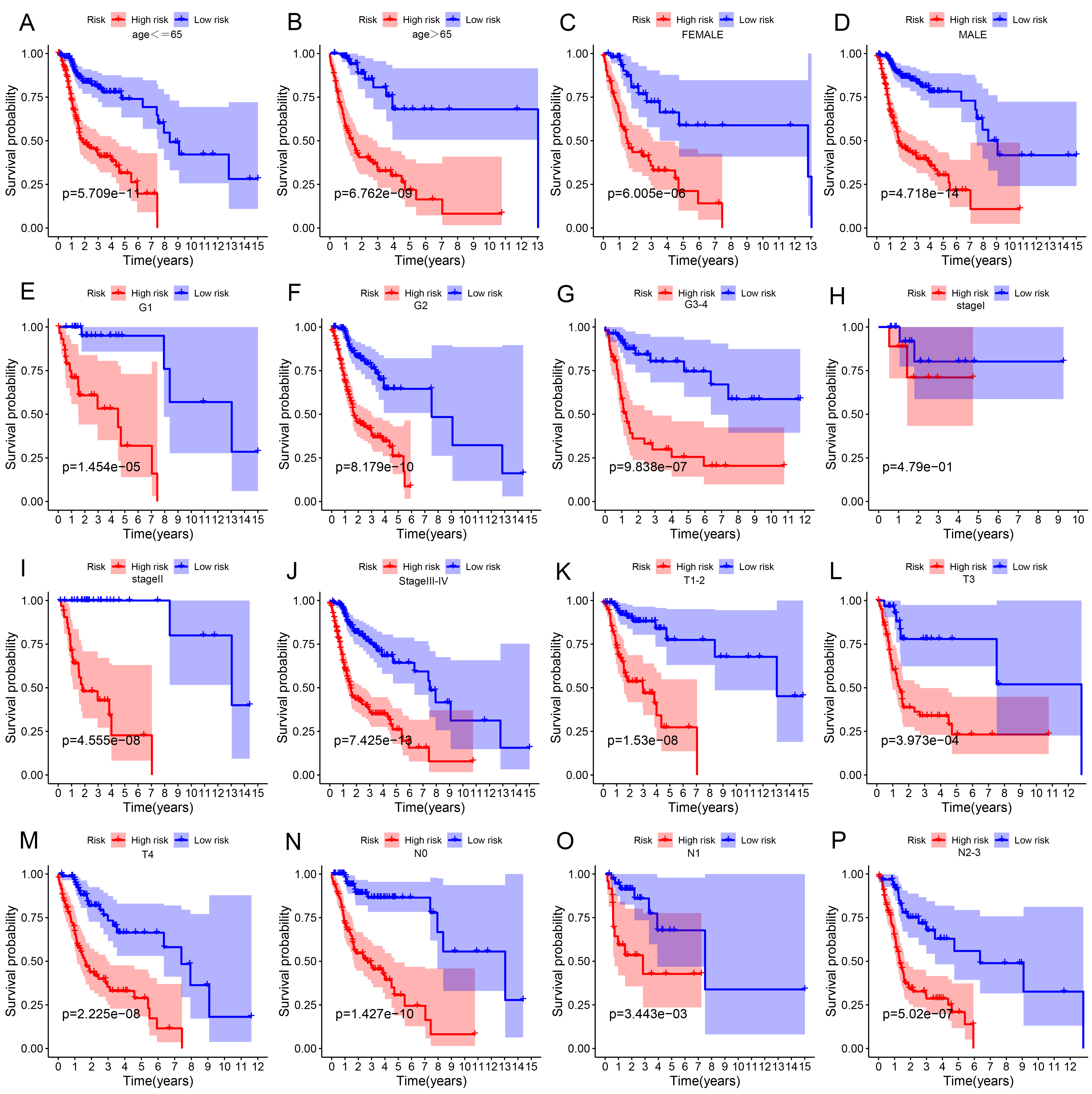

Supplement: Supplementary file 2 — Additional file 2: Fig. S2. Survival correlation analysis in different subtypes of each clinical feature. The changes of survival probability with time in different subtypes of different clinical characteristics in high-risk and low-risk groups. [file 12957_2023_3066_MOESM2_ESM.jpg]

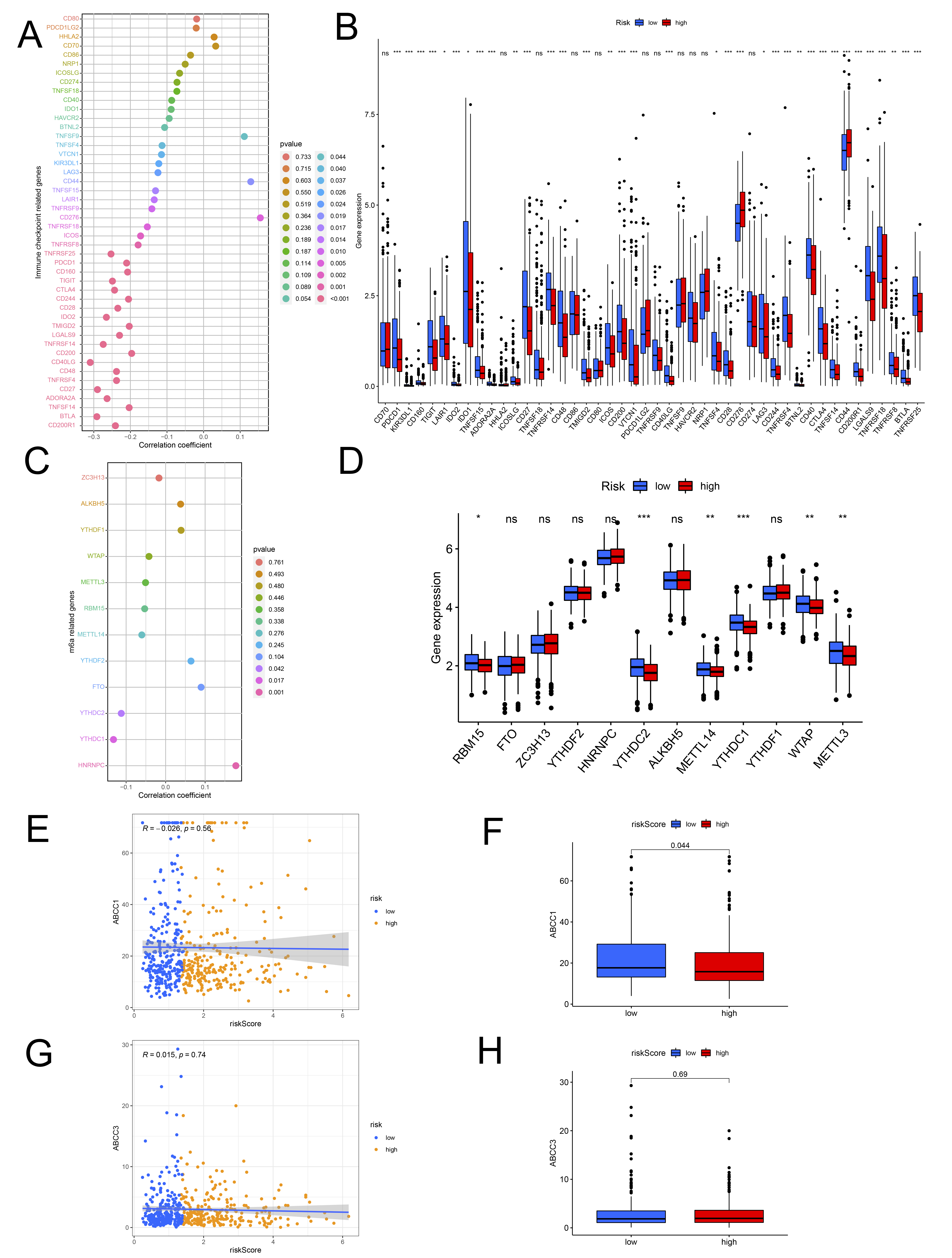

Supplement: Supplementary file 4 — Additional file 4: Fig. S4. The relevance analysis between risk score and ICIs-related genes/m6a-related / multidrug resistance genes' expression level and the comparison of these parameters in different risk groups. (A, B) The expression level of ICIs-related genes. (C, D) The expression level of m6a-related genes. (E, F) The expression level of ABCC1. (G, H) The expression level of ABCC3. [file 12957_2023_3066_MOESM4_ESM.jpg]

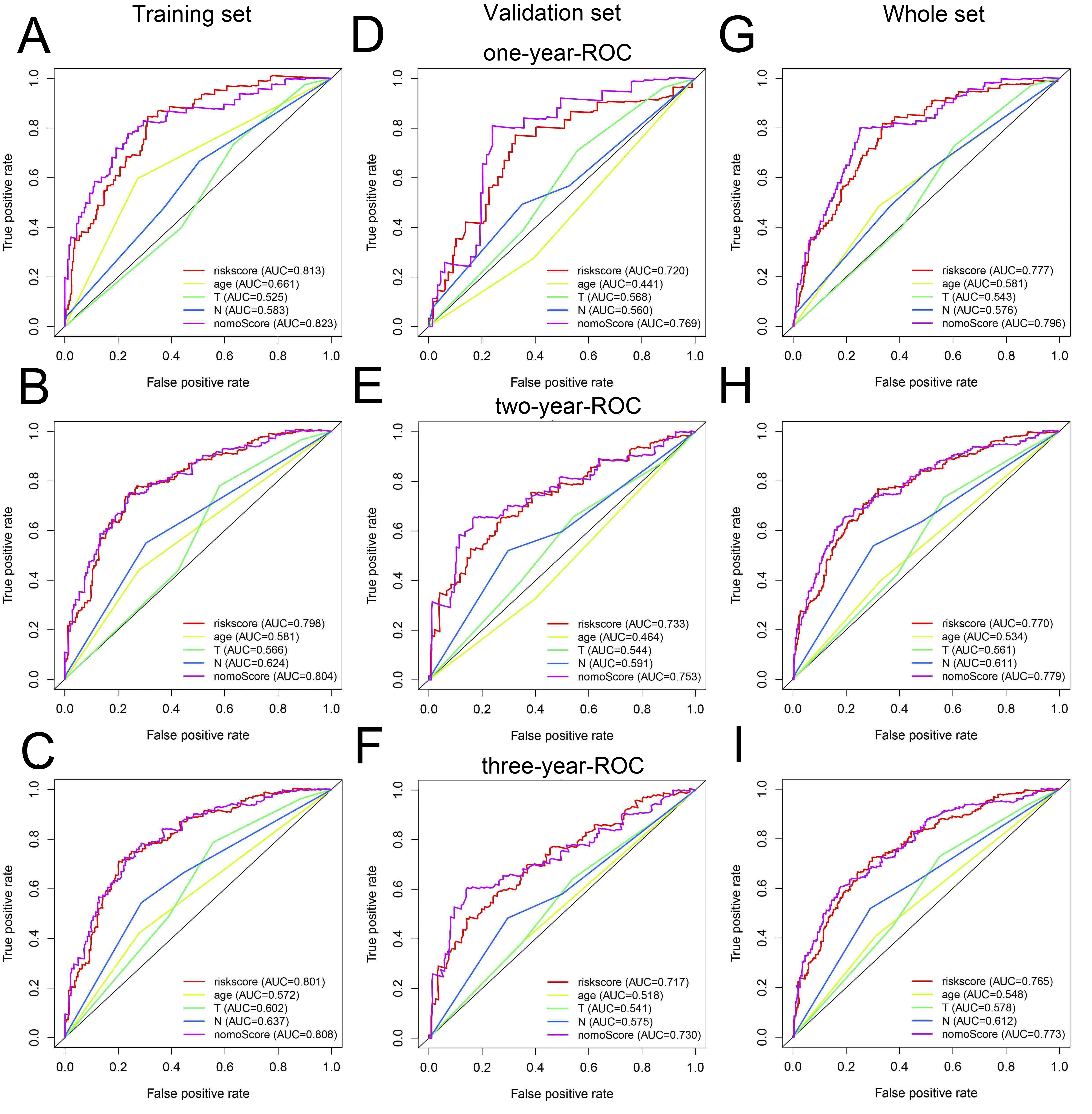

Supplement: Supplementary file 5 — Additional file 5: Fig. S5. The multi-factor ROC curves were used to confirm the Nomogram with the optimal predictive performance. 1-, 2-, and 3-years of multi-factor ROC curves based on three sets: (A-C) The training set. (D-F) The test set. (G-I) The whole set. [file 12957_2023_3066_MOESM5_ESM.jpg]

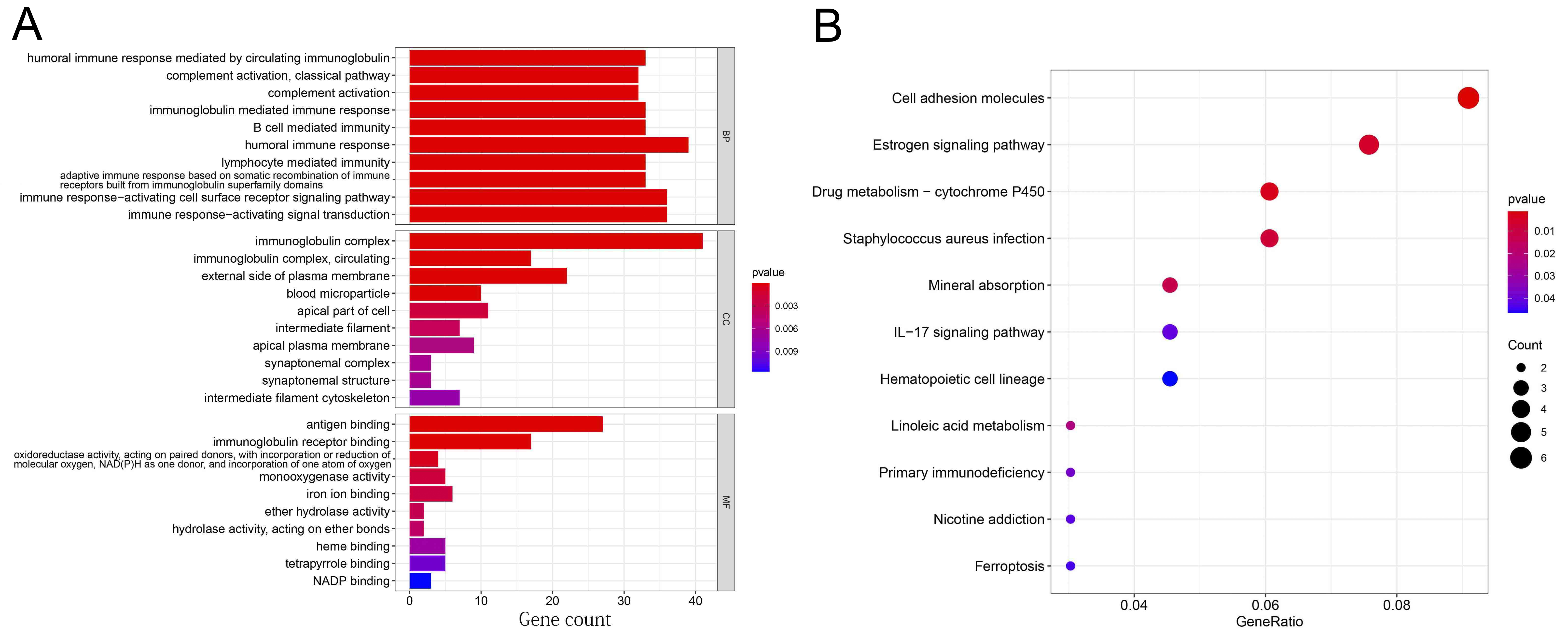

Supplement: Supplementary file 6 — Additional file 6: Fig. S6. Results of GO and KEGG enrichment analysis. (A) BPs, CCs, and MFs that are strikingly enriched by GO. (B) Pathways are strikingly enriched by KEGG. Different colors of circles and rectangles represent different significances. The size of the circle corresponds to the different ratios of DEGs enriched by each function to the total number of DEGs. [file 12957_2023_3066_MOESM6_ESM.jpg]
